# A Mild Synthesis of Bicyclic Alkoxyoxazolium Salts from Proline and Pipecolic Acid Derivatives

**DOI:** 10.1002/ejoc.201900985

**Published:** 2019-08-08

**Authors:** Eleonora Spinozzi, Adriano Bauer, Nuno Maulide

**Affiliations:** ^1^ Institute of Organic Chemistry University of Vienna Währinger Straße 38 1090 Vienna Austria

**Keywords:** Amide activation, Oxazolium, Chemoselectivity, Cycloaddition, Synthesis design

## Abstract

A regio‐ and chemoselective preparation of bicyclic alkoxyoxazolium salts from amide derivatives of proline and pipecolic acid by electrophilic amide activation is reported. Mechanistic NMR experiments suggest an unusual role for the base and highlight the effect of substitution pattern of the substrates.

## Introduction

**Scheme 1:**
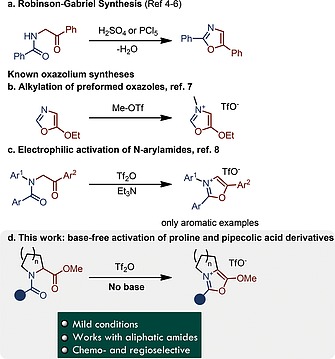
(a–c) Prior art for the synthesis of oxazolium salts and (d) this work.

The high abundance of carboxamides^1^ in combination with their distinct, mild nucleophilic properties^2^ makes them an interesting target for the investigation of organic reactions. Already in the 19^th^ century it was observed that primary amides react readily with dehydrating agents such as PCl_5_ or concentrated sulfuric acid to give nitriles.^3^ This resulted in an early recognition that the poor electrophilicity of the carbonyl‐carbon of carboxamides can be readily enhanced by electrophilic activation.^2^ Robinson[4a] and Gabriel[4b] discovered independently that acylated α‐amino ketones form oxazoles upon treatment with similar dehydrating agents (Scheme 1a).^5^, ^6^ The resulting oxazole products can be alkylated, although this requires highly reactive alkylating agents (Scheme 1b).^7^ Recently, the electrophilic activation of tertiary amides has been used for the synthesis of certain *N*‐aryloxazolium salts (Scheme 1c).^8^ However, that report restricted itself to the formation of oxazolium salts derived from aromatic amides.

Herein we would like to report the synthesis of bicyclic oxazolium salts based on proline‐ and pipecolic acid‐derived amides (Scheme 1d). The products display interesting reactivity and can be converted to complex structures by formal cycloaddition.[7a], 9

## Results and Discussion

**Scheme 2:**
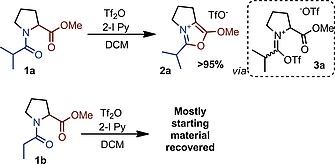
Preliminary results: oxazolium synthesis from 2‐methylpropionamide **1a** and failed reaction for propionamide** 1b**.

As part of our interest in electrophilic amide activation,^2^, ^10^ we recently investigated the suitability of proline as a chiral auxiliary for certain α‐functionalization reactions. However, when **1a** (Scheme 2) was subjected to electrophilic activation using trifluoromethanesulfonic anhydride (triflic anhydride) and 2‐iodopyridine, quantitative and fast formation of the oxazolium salt **2a** was observed.^11^ Surprisingly, slight modification of the aliphatic backbone of the amide lead to a dramatic change in reactivity: when the linear propionamide **1b** (Scheme 2) was employed instead of 2‐methylpropionamide **1a**, mostly recovered starting material was observed. Conversion of **1b** did not improve with other bases or with elevated temperature.

**Figure 1:**
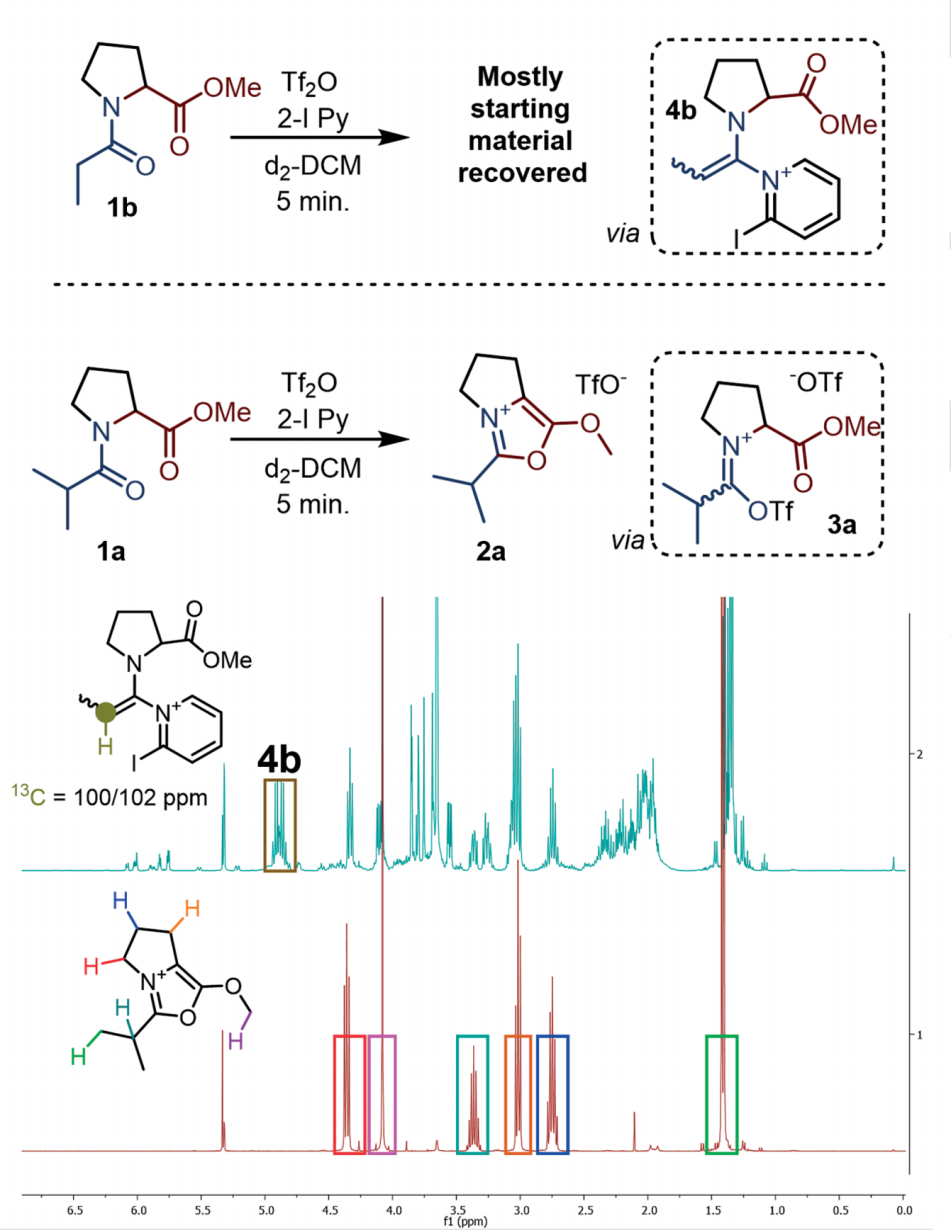
Upper NMR trace: reaction of **1b** with 2‐I Py (2.2 eq.) and Tf_2_O (1.1 eq.) in d_2_‐DCM after 5 minutes reaction time. The multiplet at 4.9 ppm is assigned to 2‐I‐pyridine adduct **4b**. Lower NMR trace: reaction of **1a** with 2‐I Py (2.2 eq.) and Tf_2_O (1.1 eq.) in d_2_‐DCM after 5 minutes reaction time. The spectrum shows a very clean formation of the desired product.

It is noteworthy that not only do the reaction mixtures of **1a** and **1b** differ considerably in appearance,^12^ but *in situ* ^1^H‐NMR spectra of those two mixtures in deuterated DCM are also strikingly different (Figure 1). As shown, while the reaction mixture of **1a** shows almost exclusively the product and the base after 5 minutes of reaction time, the spectrum of **1b** under the same reaction conditions is much more complicated.

The successful reaction of **1a** most likely proceeds *via* interception of the activated amide **3a** (Figure 1) by the pendant ester moiety, with a subsequent (formal) elimination of trifluoromethanesulfonic acid (triflic acid).

In the case of **1b**, although trace amounts of the product can be detected, other species dominate the spectrum. The species characterized by a signal at *ca*. 5.0 ppm, labelled as **4b** (Figure 1), appears to be the main compound. The structure of **4b** has been assigned as a 2‐iodopyridine adduct by 2D‐NMR analysis (see Supporting Information for details) and is a common intermediate in the electrophilic amide activation regime.[10d]

Interestingly, not even traces of an analogous species derived from **1a** can be detected by ^1^H NMR under the same reaction conditions. These findings suggest that, in the case of **1a**, limited accessibility of the α‐proton (by virtue of the α‐substituent) slows deprotonation and formation of enamine‐type adducts; this in turn is likely to greatly favor oxazolium formation in these systems.

**Scheme 3:**
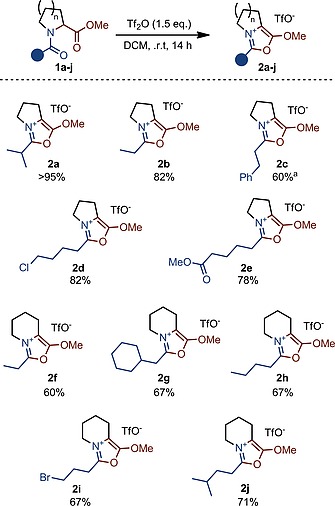
See Supporting Information for detailed reaction conditions. Yield refers to the pure isolated product.^[a]^ 2.5 equiv. of Tf_2_O used.

**Figure 2:**
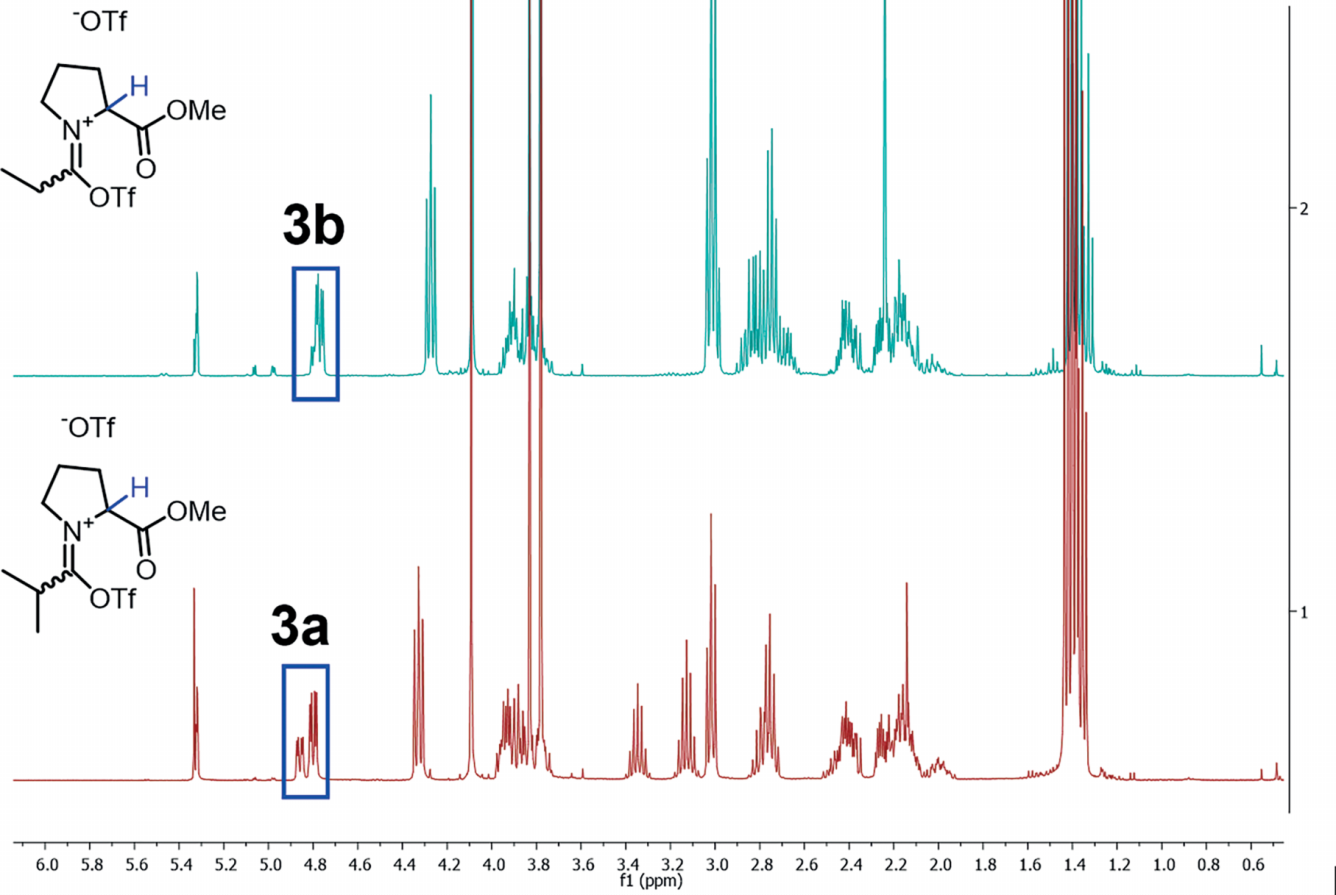
Upper NMR trace: reaction of **1b** with Tf_2_O (1.1 eq.) in d_2_‐DCM after 5 minutes reaction time. The multiplet at 4.8 ppm is assigned to the cation **3b** (the only other species present in considerable amounts is the desired oxazolium product). Lower NMR trace: reaction of **1a** with Tf_2_O (1.1 eq.) in d_2_‐DCM after 5 minutes reaction time.

These observations would suggest that omitting the base might allow oxazolium formation even in “unbranched” substrates such as **1b**. In the event, such a simple modification (Scheme 3) indeed led to the expected oxazolium **2b** in 82 % yield.^13^ The ^1^H NMR spectrum of **1b** in d_2_‐DCM after 5 minutes reaction time (Figure 2) now shows clean product formation, with a species assigned as intermediate **3b** as the major compound in the mixture. The conspicuous absence of vinylic C‐H resonances, indicative of a slower deprotonation, should be noted.

Several other bicyclic, alkoxyoxazolium salts could be synthesized through this procedure in good to excellent yields (Scheme 3). Halogens such as chloride (**2d**) or bromide (**2i**) were tolerated. Pleasingly, an additional ester on the aliphatic chain does not interfere in the process.[2e] It is also noteworthy that a phenyl ring in proximity to the activated amide (**2c**) does not trigger Friedel‐Crafts reactivity. The reaction was also found to be amenable to pipecolic acid derivatives (**2f**–**2j**).

**Scheme 4:**
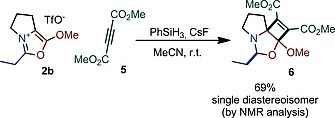
Possible further transformation of one oxazolium salt.

We noted that the products structurally resemble münchnones^14^ to some extent. The latter are well‐known for their ready participation in interesting (3+2) cycloadditions.^14^ In the event, we achieved a reductive formal [2+2]‐cycloaddition of oxazolium salt **2b** with dimethyl acetylenedicarboxylate (DMDA) leading to product **6b** in good yield and as a single diastereoisomer (Scheme 4).^9^ The NOESY NMR spectrum of compound **6** is consistent with the stereochemistry shown in Scheme 4.

## Conclusion

Herein we reported that bicyclic, alkoxyoxazolium salts can be readily prepared from simple proline‐ and pipecolic acid derivatives. Mechanistic experiments highlighted a deleterious role for the base. The products lend themselves to synthetic elaboration by cycloaddition reactions.

## Supporting information

Supporting Information
