# Supplementary material for: A Mild Synthesis of Bicyclic Alkoxyoxazolium Salts from Proline and Pipecolic Acid Derivatives
Source: European J Org Chem. 2019 Aug 8;2019(31-32):5230–3. doi: 10.1002/ejoc.201900985 (PMC6771909; doi:10.1002/ejoc.201900985)
Supplement: Supplementary file 1 — Supporting Information [file EJOC-2019-5230-s001.pdf]

European Journal of Organic Chemistry

Supporting Information

A Mild Synthesis of Bicyclic Alkoxyoxazolium Salts from Proline and Pipecolic Acid Derivatives

Eleonora Spinozzi, Adriano Bauer, and Nuno Maulide*

ejoc201900985-sup-0001-SupMat.pdf

Table of Contents

I. Preparation of amide starting material 2
General procedure A 2
II. Synthesis of oxazolium salts 2
Preliminary result 2
General procedure B 2
General procedure C 3
III. Synthesis of cyclobutene **6b** *via* reductive cycloaddtion 3
IV. NMR experiments 3
General procedure D 3
General procedure E 4
V. Characterization 6
VI. Spectra 11

General information

Unless otherwise stated, all glassware was flame-dried before use and all reactions were performed under an atmosphere of argon. All solvents were distilled from appropriate drying agents prior to use. All reagents were used as received from commercial suppliers unless otherwise stated. Reaction progress was monitored by thin layer chromatography (TLC) performed on aluminium plates coated with silica gel F254 with 0.2 mm thickness. Chromatograms were visualized by fluorescence quenching with UV light at 254 nm or by staining using potassium permanganate. Flash column chromatography was performed using silica gel 60 (230-400 mesh, Merck and co.). Neat infrared spectra were recorded using a Perkin-Elmer Spectrum 100 FT-IR spectrometer. Wavenumbers (νmax) are reported in cm^{-1}. Mass spectra were obtained using a Finnigan MAT 8200 or (70 eV) or an Agilent 5973 (70 eV) spectrometer, using electrospray ionization (ESI). All ^{1}H NMR and ^{13}C NMR spectra were recorded using a Bruker AV-400, AV-600 or AV-700 spectrometer at 300K. Chemical shifts were given in parts per million (ppm, δ), referenced to the solvent peak of CDCl3, defined at δ = 7.26 ppm (^{1}H-NMR) and δ = 77.16 (^{13}C NMR). Coupling constants are quoted in Hz (J). ^{1}H NMR splitting patterns were designated as singlet (s), doublet (d), triplet (t), quartet (q). Splitting patterns that could not be interpreted or easily visualized were designated as multiplet (m) or broad (br).

I. Preparation of amide starting material

General procedure A

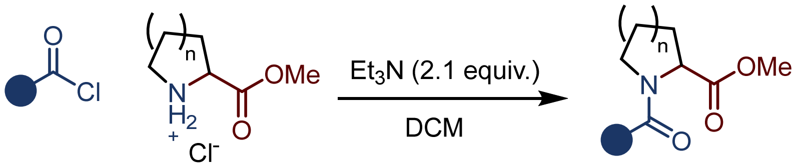

To a solution of the amine hydrochloride (1.0 equiv.) and triethylamine (2.1 equiv.) in DCM (0.5 M) at r.t. a solution of the corresponding acyl chloride (1.0 equiv.) in DCM was added dropwise and the resulting reaction mixture was stirred 1h at the same temperature. After this time, a saturated aqueous solution of sodium bicarbonate was added and the biphasic system was separated. The aqueous phase was extracted with DCM (3 ×), the organic phases were combined and then washed with HCl (1 N). The organic layer was then dried over anhydrous sodium sulfate. The dried solution was filtered and concentrated under reduced pressure. The resulting crude material was purified by silica gel column chromatography (typically 30:70 - heptanes: EtOAc) to afford the desired compound.

II. Synthesis of oxazolium salts

Preliminary result

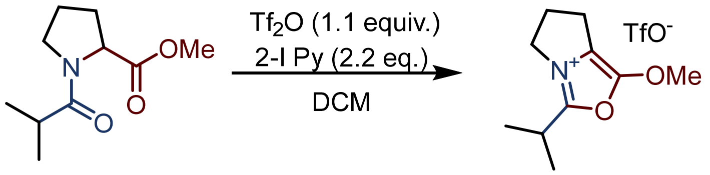

To a solution of the amide **1a** (1.0 equiv., 0.2 mmol) in DCM (0.2 M) at r.t. was added 2-iodo pyridine (2.2 equiv., 0.44 mmol, 47 µL) and the reaction was cooled to 0 °C. Trifluoromethanesulfonic anhydride (1.1 equiv., 0.22 mmol, 37 µL) was added dropwise and the reaction was stirred for 10 minutes at the same temperature. The reaction was quenched with Water, extracted with DCM and the combined organic layer was dried over anhydrous Na_2SO_4, volatiles were removed and the crude product was purified by column chromatography first EtOAc (100% - *ca.* 40 mL) and then isopropanol (100%), to obtain the product typically as a yellow oil (see characterization for appearance and yields).

General procedure B

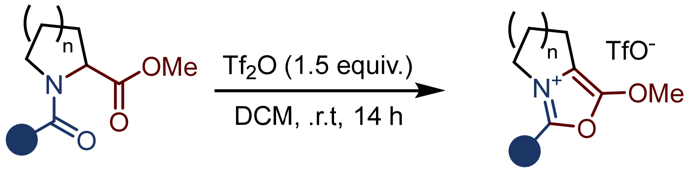

To a solution of the amide (1.0 equiv., 0.4 mmol) in DCM (0.1 M) at r.t. was added trifluoromethanesulfonic anhydride (1.5 equiv., 0.6 mmol) and the reaction was stirred 14 h at the same temperature. After this time DCM saturated with water (3 mL) was added to the reaction mixture and the reaction was stirred for 1 h at the same temperature. Then the organic phase was dried over anhydrous magnesium sulfate, filtered and concentrated under reduced pressure. The resulting crude material was purified by silica gel column chromatography; first EtOAc (100% - *ca.* 40 mL) and then isopropanol (100%), to obtain the product typically as a yellow oil (see characterization for appearance and yields).

General procedure C

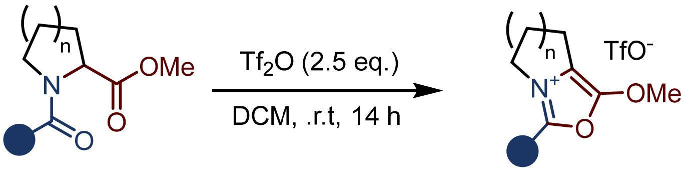

To a solution of the amide (1.0 equiv., 0.4 mmol) in DCM (0.1 M) at r.t. was added trifluoromethanesulfonic anhydride (2.5 equiv., 1.0 mmol) and the reaction was stirred 14 h at the same temperature. After this time DCM saturated with water (3 mL) was added to the reaction mixture and the reaction was stirred for 1 h at the same temperature. Then the organic phase was dried over anhydrous magnesium sulfate, filtered and concentrated under reduced pressure. The resulting crude material was purified by silica gel column chromatography; first EtOAc (100% - *ca.* 40 mL) and then isopropanol (100%), to obtain the product typically as a yellow oil (see characterization for appearance and yields).

III. Synthesis of cyclobutene **6** *via* reductive cycloaddtion

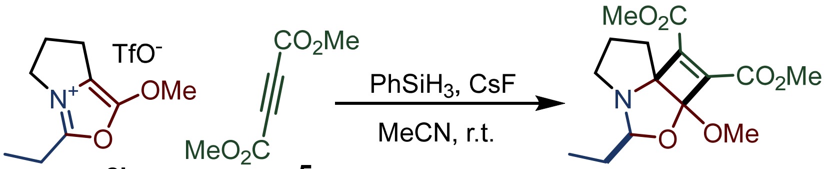

2b **5** **6**

Commercially available cesium fluoride was dried at 100 °C for 2 h under vacuum.The oxazolium salt (63.5 mg, 0.2 mmol) was dissolved in dry acetonitrile (3 mL) at r.t. Afterwards, phenylsilane (37 µL, 0.3 mmol, 1.5 equiv.) and DMAD (81 µL, 0.66 mmol, 3.3 equiv.), were added and this mixture was added to a suspension of anhydrous cesium fluoride in acetonitrile (4 mL). After 14 h of stirring at room temperature, the solvent was removed under reduced pressure and flash chromatography (EtOAc: Hept- 4:6) of the crude gave the desired product as an orange oil (43.2 mg, 69%).

IV. NMR experiments

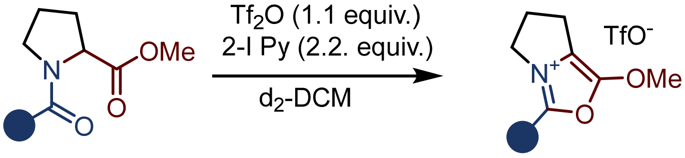

General procedure D

To a solution of the amide (1.0 equiv., 0.2 mmol) in freshly distilled d_2-DCM (0.2 M) at r.t. was added 2-iodo pyridine (2.20 equiv., 0.44 mmol, 47 µL) and the reaction was cooled to 0 °C. Trifluoromethanesulfonic anhydride (1.1 equiv., 0.22 mmol, 37 µL) was added dropwise and the reaction was stirred for 5 minutes at the same temperature. Then half of the volume of the reaction mixture was taken out and submitted to NMR analysis.

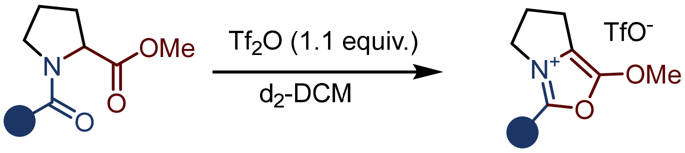

General procedure E

To a solution of the amide (1.0 equiv., 0.2 mmol) in freshly distilled d_2-DCM (0.2 M) at 0 °C was added trifluoromethanesulfonic anhydride (1.1 equiv., 0.22 mmol, 37 µL) dropwise and the reaction was stirred for 5 minutes at the same temperature. Then half of the volume of the reaction mixture was taken out and submitted to NMR analysis.

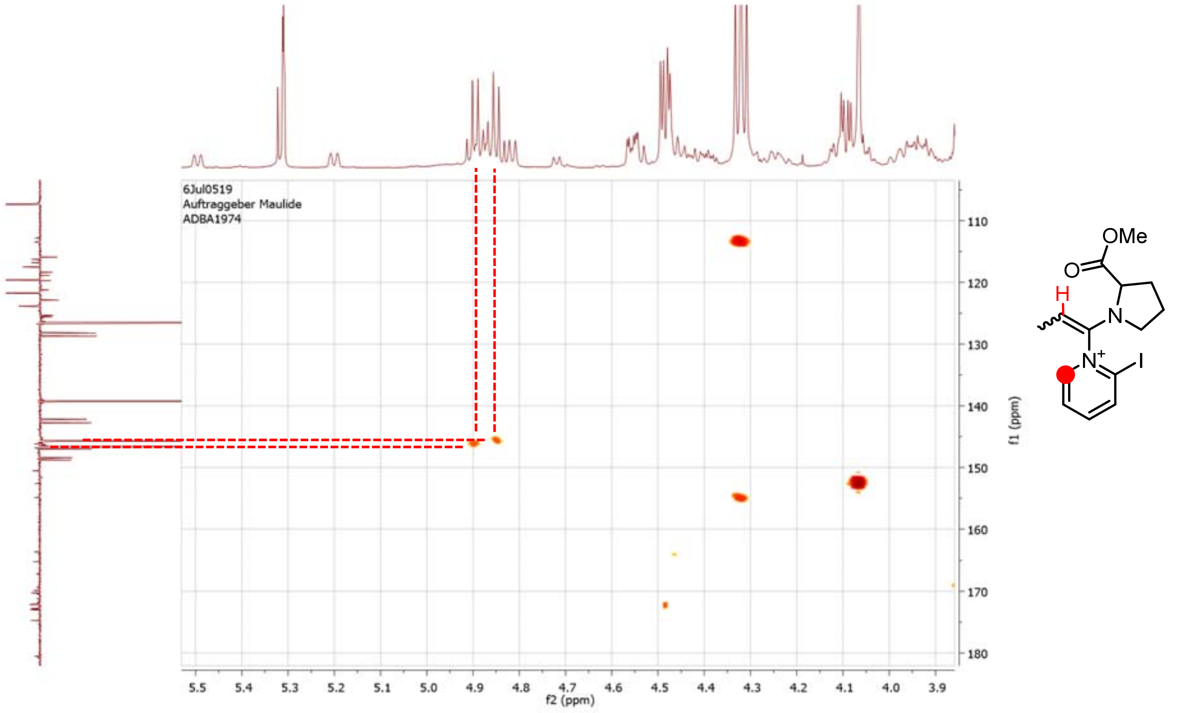

HMBC coupling of the highlighted ^{13}C with the vinylic proton.

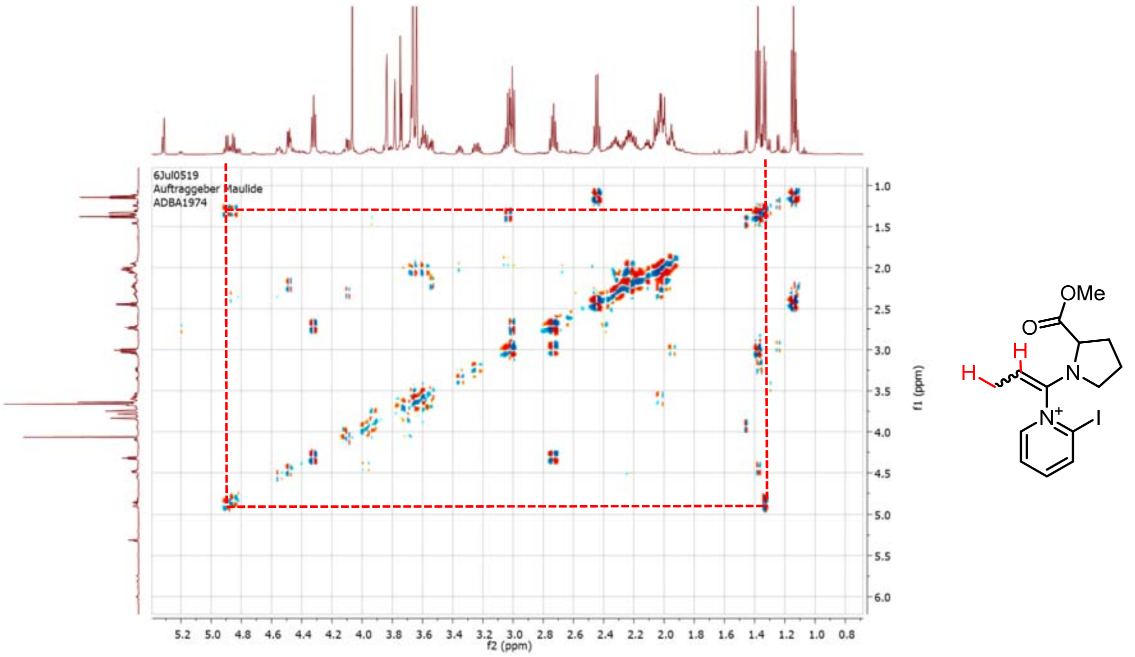

^{1}H-^{1}H COSY coupling between the allylic proton (doublet) and the vinylic proton (quadruplet)

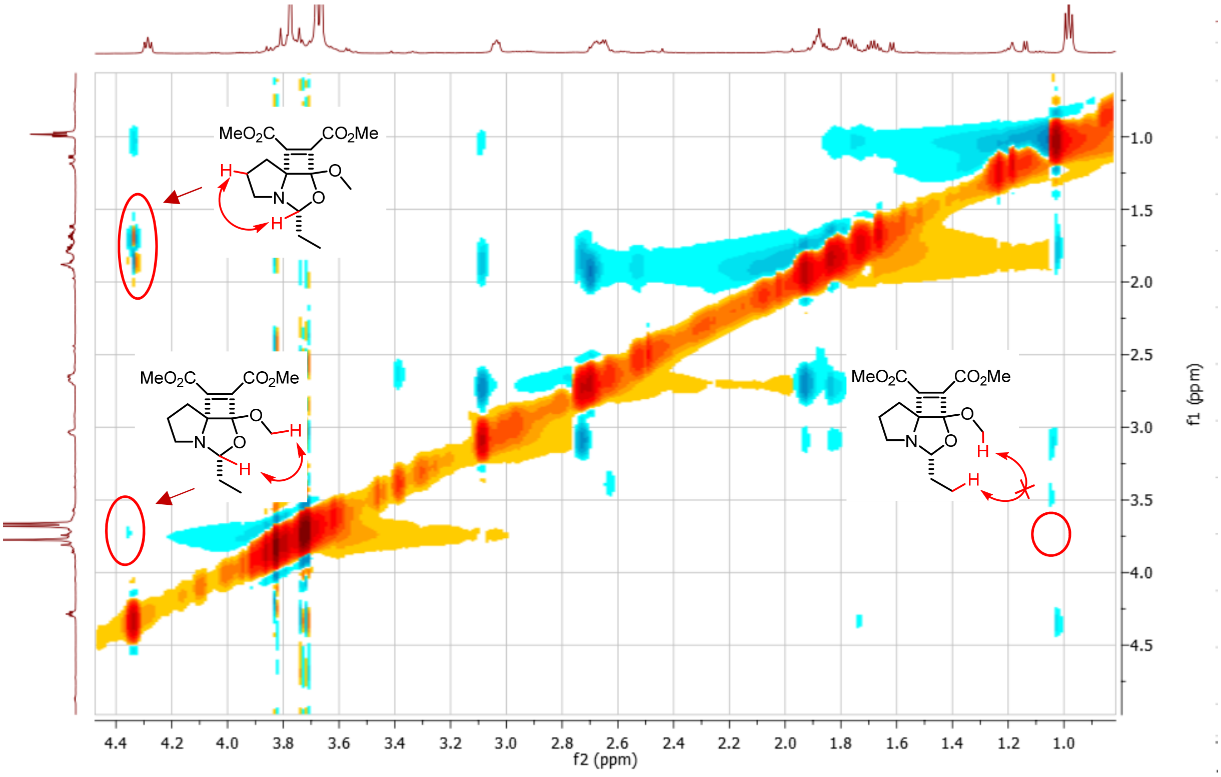

NOESY coupling of the highlighted ^{1}H in the tricyclic product **6**. On the right a missing coupling which would be expected for the other diastereoisomer.

V. Characterization

Methyl isobutyrylprolinate (1a)

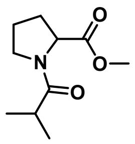

Prepared according to general procedure A, quantitative yield. 1a is a known compound (*Tetrahedron Letters* **2012,** *53,* 4413 – 4417)

Methyl propionylprolinate (1b)

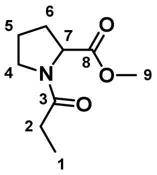

Prepared according to general procedure A, quantitative yield; **^{1}H NMR (400 MHz, $CDCl_3$)** δ 4.47 (dd, *J* = 8.5, 3.9 Hz, 1H; H7), 4.37 (dd, *J* = 8.6, 2.7 Hz, 1H; H7), 3.73 (s, 3H; H9), 3.70 (s, 3H; H9), 3.66 – 3.54 (m, 2H; H4), 3.52 – 3.42 (m, *J* = 9.8, 6.9 Hz, 2H; H4), 2.39 – 2.23 (m, *J* = 12.4, 6.2 Hz, 2H; H6), 2.20 – 1.86 (m, 4H; H2, H5), 1.13 (t, *J* = 7.5 Hz, 3H; H1). **^{13}C NMR (101 MHz, $CDCl_3$)** δ 173.14 (C3), 172.71 (C8), 58.69 (C7), 52.23 (C9), 46.93 (C4), 29.33 (C6), 27.71 (C2), 24.90 (C5), 8.76(C1). **ATR-FTIR** (cm^{-1})**:** 2977, 2953, 2878, 1739, 1641, 1422, 1365, 1342, 1308, 1280, 1195, 1171, 1095, 1081, 1043, 1029, 999, 937, 916, 873, 816, 733, 669, 595. **HRMS (ESI+):** exact mass calculated for $[M+Na]^+$ ($C_9H_{15}NNaO_3$) requires *m/z* 208.0950, found *m/z* 208.0947.

Methyl (3-phenylpropanoyl)prolinate (1c)

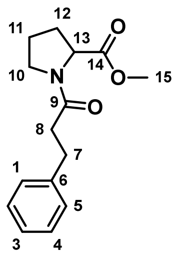

Prepared according to general procedure A, quantitative yield; **^{1}H NMR (400 MHz, $CDCl_3$)** δ 7.31 – 7.26 (m, 2H; H2, H4), 7.24 – 7.16 (m, 3H; H1, H5, H3), 4.49 (dd, *J* = 8.5, 3.8 Hz, 1H; H13), 4.25 (dd, *J* = 7.9, 3.2 Hz, 1H; H13), 3.73 (s, 3H; H15), 3.70 (s, 3H; H15), 3.60 – 3.51 (m, *J* = 10.2, 8.0, 5.2 Hz, 2H; H10), 3.42 – 3.35 (m, 2H; H10), 2.98 (t, *J* = 7.9 Hz, 2H; H7), 2.70 – 2.41 (m, 2H; H8), 2.20 – 1.84 (m, 4H; H12, H11). **^{13}C NMR (101 MHz, $CDCl_3$)** δ 173.05 (C9), 171.18 (C14), 141.50 (C6), 128.58 (C2,C4), 126.22 (C1, C5, C3), 58.76 (C13), 52.30 (C15), 47.06 (C10), 36.57 (C8), 30.93 (C7), 29.34 (C12), 24.89 (C11). **ATR-FTIR** (cm^{-1})**:** 3026, 2952, 2878, 1739, 1642, 1603, 1496, 1420, 1364, 1341, 1280, 1244, 1195, 1171, 1095, 1078, 1057, 1029, 951, 911, 818, 788, 751, 699, 624, 595. **HRMS (ESI+):** exact mass calculated for $[M+Na]^+$ ($C_{15}H_{19}NNaO_3$) requires *m/z* 284.1263, found *m/z* 284.1258.

Methyl (5-chloropentanoyl)prolinate (1d)

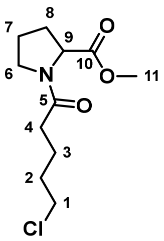

Prepared according to general procedure A, quantitative yield; **^{1}H NMR (400 MHz, $CDCl_3$)** δ 4.47 (dd, *J* = 8.6, 4.0 Hz, 1H; H9), 4.39 (dd, *J* = 8.5, 2.7 Hz, 1H; H9), 3.75 (s, 3H; H11), 3.71 (s, 3H; H11), 3.67 – 3.44 (m, 4H; H1, H6), 2.45 – 2.26 (m, 2H; H8), 2.22 – 1.92 (m, 4H; H4,H7), 1.90 – 1.74 (m, 4H; H2, H3). **^{13}C NMR (101 MHz, $CDCl_3$)** δ 173.01(C5), 171.36 (C10), 58.74 (C9), 52.28 (C11), 47.10 (C6), 44.89 (C1), 33.53 (C4), 32.16 (C3), 29.32 (C8), 24.93 (C7), 22.04 (C2). **ATR-FTIR** (cm^{-1})**:** 2953, 2876, 1741, 1641, 1421, 1363, 1326, 1277, 1195, 1171, 1095, 1043, 998, 874, 830, 745, 722, 645. **HRMS (ESI+):** exact mass calculated for $[M+Na]^+$ ($C_{11}H_{18}ClNNaO_3$) requires *m/z* 270.0873, found *m/z* 270.0869.

Methyl (6-methoxy-6-oxohexanoyl)prolinate (1e)

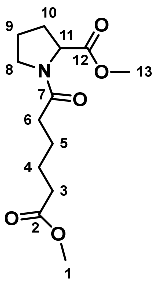

Prepared according to general procedure A, quantitative yield; **^{1}H NMR (400 MHz, $CDCl_3$)** δ 4.46 (dd, *J* = 8.5, 4.0 Hz, 1H; H11), 4.38 (dd, *J* = 8.5, 2.7 Hz, 1H; H11), 3.75 (s, 3H; H13), 3.70 (s, 3H; H13), 3.64 (s, 3H; H1), 3.62 – 3.55 (m, 2H; H8), 3.52 – 3.43 (m, 2H; H8), 2.36 – 2.26 (m, *J* = 9.5, 7.1, 3.1 Hz, 4H; H3, H10), 2.20 – 1.92 (m, 4H; H6, H9), 1.70 – 1.65 (m, 4H; H4, H5). **^{13}C NMR (101 MHz, $CDCl_3$)** δ 174.05 (C7), 173.05 (C2), 171.52 (C12), 58.71 (C11), 52.25 (C13), 51.59 (C1), 47.07 (C8), 34.11 (C6), 33.98 (C3), 29.32 (C10), 24.92 (C5), 24.70 (C9), 24.16 (C4). **ATR-FTIR** (cm^{-1})**:** 2953, 2878, 1732, 1645, 1421, 1364, 1324, 1262, 1239, 1194, 1170, 1095, 1044, 1002, 912, 876, 831, 765, 734, 672. **HRMS (ESI+):** exact mass calculated for $[M+Na]^+$ ($C_{13}H_{21}NNaO_5$) requires *m/z* 294.1317, found *m/z* 294.1317.

Methyl 1-propionylpiperidine-2-carboxylate (1f)

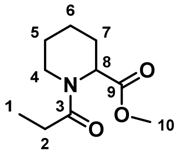

Prepared according to general procedure A, quantitative yield; **^{1}H NMR (400 MHz, $CDCl_3$)** δ 5.38 (d, *J* = 5.4 Hz, 1H; H8), 4.56 (d, *J* = 6.8 Hz, 1H; H8), 3.74 (s, 3H; H10), 3.72 (d, *J* = 1.6 Hz, 1H; H4), 3.70 (s, 3H; H10), 3.22 (td, *J* = 13.0, 3.0 Hz, 1H; H4), 2.42 – 2.18 (m, *J* = 15.9, 13.7, 9.6, 5.9 Hz, 3H; H7, H2), 1.75 – 1.55 (m, *J* = 13.5, 11.2, 8.5, 3.5 Hz, 3H; H2, H5), 1.49 – 1.27 (m, 2H; H6), 1.14 (t, *J* = 7.4 Hz, 3H; H1). **^{13}C NMR (101 MHz, $CDCl_3$)** δ 173.94 (C3), 172.17 (C9), 56.05 (C8), 51.95 (C10), 43.33 (C4), 26.76 (C7), 26.73 (C2), 25.46 (C5), 21.11 (C6), 9.38 (C1). **ATR-FTIR** (cm^{-1})**:** 2940, 2861, 1736, 1644, 1420, 1376, 1312, 1241, 1201, 1160, 1144, 1111, 1076, 1022, 1006, 928, 868, 858, 821, 782, 743, 625. **HRMS (ESI+):** exact mass calculated for [M+Na]$^+$ ($C_{10}H_{17}NNaO_3$) requires *m/z* 222.1106, found *m/z* 222.1105.

Methyl 1-(2-cyclohexylacetyl)piperidine-2-carboxylate (1g)

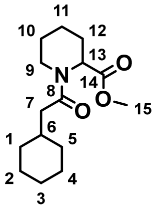

Prepared according to general procedure A, quantitative yield; **^{1}H NMR (400 MHz, $CDCl_3$)** δ 5.42 (d, *J* = 5.3 Hz, 1H; H13), 4.58 (d, *J* = 12.5 Hz, 1H; H13), 3.83 – 3.76 (m, *J* = 13.7, 1.5 Hz, 1H; H9), 3.75 (s, 3H; H15), 3.71 (s, 3H; H15), 3.21 (td, *J* = 13.0, 2.9 Hz, 1H; H9), 2.35 – 2.10 (m, 3H; H12, H6), 1.84 – 0.89 (m, 16H; H7, H10, H11, H1, H2, H3, H4, H5). **^{13}C NMR (101 MHz, $CDCl_3$)** δ 172.64 (C8), 172.12 (C14), 56.44 (C13), 51.88 (C15), 43.90 (C9), 41.01 (C7), 35.16 (C1), 33.50 (C5), 33.45 (C6), 26.78 (C12), 26.43 (C3), 26.34 (C10), 26.30 (C2), 25.6 (C4), 21.14 (C11). **ATR-FTIR** (cm^{-1})**:** 2922, 2851, 1738, 1641, 1470, 1416, 1368, 1354, 1337, 1320, 1239, 1202, 1158, 1144, 1125, 1107, 1021, 996, 971, 868, 857, 821, 785, 760, 733, 701, 622. **HRMS (ESI+):** exact mass calculated for [M+Na]$^+$ ($C_{15}H_{25}NNaO_3$) requires *m/z* 290.1732, found *m/z* 290.1728.

Methyl 1-pentanoylpiperidine-2-carboxylate (1h)

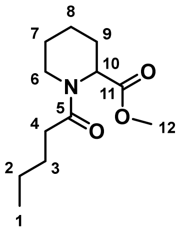

Prepared according to general procedure A, quantitative yield; **^{1}H NMR (400 MHz, $CDCl_3$)** δ 5.40 (d, *J* = 5.3 Hz, 1H; H10), 4.57 (d, *J* = 12.4 Hz, 1H; H10), 3.79 – 3.76 (m, *J* = 1.3 Hz, 1H; H6), 3.75 (s, 3H; H12), 3.71 (s, 3H; H12), 3.23 (td, *J* = 13.0, 2.9 Hz, 1H; H6), 2.44 – 2.18 (m, 3H; H9, H4), 1.77 – 1.54 (m, 5H; H4, H7, H8), 1.50 – 1.23 (m, 4H; H3, H2), 0.93 (t, *J* = 7.3 Hz, 3H; H1). **^{13}C NMR (101 MHz, $CDCl_3$)** δ 173.39 (C5) , 172.18 (C11), 56.26 (C10), 51.91 (C12), 43.60 (C6), 33.36 (C4), 27.38 (C3), 26.77 (C9), 25.57 (C7), 22.66 (C2), 21.15 (C8), 14.02 (C1). **ATR-FTIR** (cm^{-1})**:** 2934, 2863, 1738, 1643, 1419, 1259, 1202, 1159, 1145, 1114, 1104, 1023, 868, 822, 787, 733, 702. **HRMS (ESI+):** exact mass calculated for [M+Na]$^+$ ($C_{12}H_{21}NNaO_3$) requires *m/z* 250.1419, found *m/z* 250.1417.

Methyl 1-(4-bromobutanoyl)piperidine-2-carboxylate (1i)

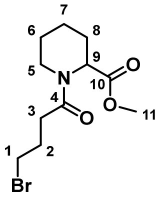

Prepared according to general procedure A, quantitative yield; **^{1}H NMR (400 MHz, $CDCl_3$)** δ 5.37 (d, *J* = 5.3 Hz, 1H; H9), 4.62 (d, *J* = 4.7 Hz, 1H; H9), 3.77 (s, 3H; H11), 3.73 (s, 3H; H11), 3.64 (t, *J* = 6.2 Hz, 2H; H5) 3.52 (t, *J* = 6.2 Hz, 2H; H5), 3.25 (td, *J* = 13.0, 2.8 Hz, 1H; H1), 2.71 – 2.08 (m, 5H; H1, H8, H2), 1.78 – 0.70 (m, 6H; H3, H6, H7). **^{13}C NMR (101 MHz, $CDCl_3$)** δ 172.02 (C4), 171.93 (C10), 56.15 (C9), 52.14 (C11), 43.50 (C5), 34.01 (C1), 31.44 (C3), 28.12 (C2), 26.72 (C8), 25.44 (C6), 21.08 (C7). **ATR-FTIR** (cm^{-1})**:** 2946, 2863, 1737, 1641, 1431, 1243, 1206, 1161, 1144, 1110, 1079, 1018, 995, 869, 822,, 732, 701. **HRMS (ESI+):** exact mass calculated for [M+Na]$^+$ ($C_{11}H_{18}BrNNaO_3$) requires *m/z* 314.0368, found *m/z* 314.0360.

Methyl 1-(4-methylpentanoyl)piperidine-2-carboxylate (1j)

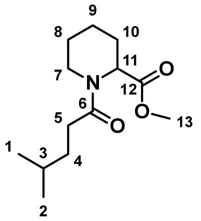

Prepared according to general procedure A, quantitative yield; **^{1}H NMR (400 MHz, $CDCl_3$)** δ 5.38 (d, *J* = 5.4 Hz, 1H; H11), 4.55 (d, *J* = 11.3 Hz, 1H; H11), 3.74 (s, 3H; H13), 3.73 – 3.72 (m, *J* = 1.5 Hz, 1H; H7), 3.70 (s, 3H; H13), 3.23 (td, *J* = 13.0, 2.9 Hz, 1H; H7), 2.36 (t, *J* = 7.9 Hz, 2H; H10), 2.28 – 2.19 (m, *J* = 9.7, 7.2, 6.0 Hz, 1H; H3), 1.74 – 1.28 (m, 8H; H9, H8, H5, H4), 0.89 (t, *J* = 7.2 Hz, 6H; H1, H2). **^{13}C NMR (101 MHz, $CDCl_3$)** δ 173.51 (C12), 172.14 (C6), 56.23 (C11), 51.88 (C13), 43.57 (C7), 34.05 (C4), 31.64 (C5), 28.01 (C3), 26.72 (C10), 25.54 (C8), 22.51 (C1), 22.47 (C2), 21.12 (C9). **ATR-FTIR** (cm^{-1})**:** 2951, 2867, 1783, 1643, 1417, 1385, 1367, 1354, 1252, 1229, 1201, 1159, 1145, 1079, 1018, 995, 951, 868, 858, 821, 782, 630, 611, 590. **HRMS (ESI+):** exact mass calculated for [M+Na]$^+$ ($C_{13}H_{23}NNaO_3$) requires *m/z* 264.1576, found *m/z* 264.1575.

3-isopropyl-1-methoxy-6,7-dihydro-5H-pyrrolo[1,2-c]oxazol-4-ium trifluoromethanesulfonate (2a)

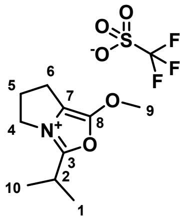

Prepared according to general procedure B as an yellow oil; 82 % yield (108.4 mg) **^{1}H NMR** (600 MHz, $CDCl_3$) δ 4.41 (t, J = 7.4 Hz, 2H, H4), 4.12 (s, 3H, H9), 3.41 (hept, J = 7.0 Hz, 1H, H2), 3.09 – 2.96 (m, 2H, H6), 2.85 – 2.74 (m, 2H, H5), 1.46 (d, J = 7.0 Hz, 7H, H1/H10). **^{13}C NMR** (151 MHz, $CDCl_3$) δ 157.55 (C8), 152.45 (C3), 114.31 (C7), 61.98 (C9), 47.99 (C4), 28.92 (C5), 28.01 (C2), 20.92 (C10 or C1), 18.77 (C10 or C1). **HRMS (ESI+)** exact mass calculated for $[M]^+$ (182.1177 found 182.1176. **ATR-FTIR** (cm^{-1}): 2984, 1704, 1613, 1453, 1359, 1252, 1225, 1152, 1084, 1030, 913, 830, 757, 716, 636.

3-Ethyl-1-methoxy-6,7-dihydro-5H-pyrrolo[1,2-c]oxazol-4-ium trifluoromethanesulfonate (2b)

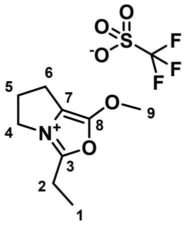

Prepared according to general procedure B as an yellow oil; 82 % yield (108.4 mg); **^{1}H NMR (400 MHz, $CDCl_3$)** δ 4.35 (t, J = 7.3 Hz, 2H; H4), 4.08 (s, 3H; H9), 3.11 – 2.95 (m, J = 14.5, 7.6 Hz, 4H; H2, H6), 2.82 – 2.68 (m, 2H; H5), 1.40 (t, J = 7.6 Hz, 3H; H1). **^{13}C NMR (101 MHz, $CDCl_3$)** δ 155.04 (C8), 152.54 (C3), 113.95 (C7), 61.78 (C9), 47.54 (C4), 28.88 (C5), 21.10 (C6), 20.52 (C2), 8.92 (C1). **ATR-FTIR** (cm^{-1})**:** 2958, 1704, 1622, 1460,1359, 1260, 1223, 1149, 1065, 1028, 974, 850, 634. **HRMS (ESI+):** exact mass calculated for $[M]^+$ ($C_9H_{14}NO_2^+$) requires m/z 168.1019, found m/z 168.1022.

1-Methoxy-3-phenethyl-6,7-dihydro-5H-pyrrolo[1,2-c]oxazol-4-ium trifluoromethanesulfonate (2c)

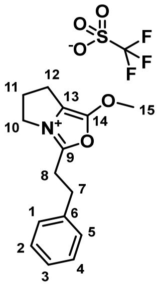

Prepared according to general procedure C as an yellow oil; 60 % yield (94,4 mg); **^{1}H NMR (400 MHz, $CDCl_3$)** δ 7.35 – 7.26 (m, 3H; H1, H5, H3), 7.16 – 7.10 (m, 2H; H2, H4), 4.10 (s, 3H; H15), 3.88 (t, J = 7.3 Hz, 2H; H10), 3.35 (t, J = 7.3 Hz, 2H; H8), 3.12 (t, J = 7.2 Hz, 2H; H7), 2.93 (t, J = 7.4 Hz, 2H; H11), 2.62 – 2.52 (m, 2H; H12).**^{13}C NMR (101 MHz, $CDCl_3$)** δ 153.35 (C14), 152.64 (C9), 138.16 (C13), 129.19 (C6), 128.38 (C2), 127.45 (C4), 113.38 (C1, C5, C3), 61.57 (C15), 47.47 (C10), 31.50 (C7), 28.84 (C8), 28.81 (C11), 21.09 (C12). **ATR-FTIR** (cm^{-1})**:** 2955, 1722, 1701,1620, 1541, 1498, 1454, 1362, 1223, 1157, 1027, 847, 755, 701, 635. **HRMS (ESI+):** exact mass calculated for $[M]^+$ ($C_{15}H_{18}NO_2^+$) requires m/z 244.1332, found m/z 244.1337.

3-(4-Chlorobutyl)-1-methoxy-6,7-dihydro-5H-pyrrolo[1,2-c]oxazol-4-ium trifluoromethanesulfonate (2d)

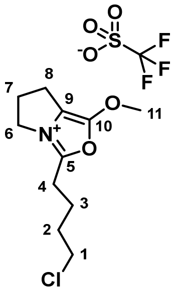

Prepared according to general procedure B as an yellow oil; 82 % yield (125 mg); **^{1}H NMR (400 MHz, $CDCl_3$)** δ 4.33 (t, J = 7.1 Hz, 2H; H6), 4.07 (s, 3H; H11), 3.58 (t, J = 6.1 Hz, 2H; H1), 3.10 – 2.91 (m, J = 17.8, 7.2 Hz, 4H; H8, H4), 2.79 – 2.66 (m, 2H; H7), 2.04 – 1.84 (m, 4H; H2, H3). **^{13}C NMR (101 MHz, $CDCl_3$)** δ 153.64 (C10), 152.58 (C5), 113.66 (C9), 61.52 (C11), 47.59 (C6), 44.11 (C1), 31.41 (C2), 28.82 (C3), 25.74 (C4), 22.20 (C7), 21.12 (C8). **ATR-FTIR** (cm^{-1})**:** 2958, 1702, 1620, 1541, 1454, 1358, 1259, 1223, 1149, 1028, 911, 892, 860, 774, 754, 714, 635. **HRMS (ESI+):** exact mass calculated for $[M]^+$ ($C_{11}H_{17}ClNO_2^+$) requires m/z 230.0942, found m/z 230.0937.

1-Methoxy-3-(5-methoxy-5-oxopentyl)-6,7-dihydro-5H-pyrrolo[1,2-c]oxazol-4-ium trifluoromethanesulfonate (2e)

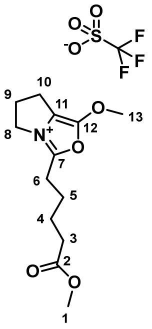

Prepared according to general procedure B as an yellow oil; 78 % yield (125.9 mg); **^{1}H NMR (400 MHz, CDCl$_3$)** δ 4.34 (t, J = 7.3 Hz, 2H; H8), 4.07 (s, 3H; H13), 3.65 (s, 3H; H1), 3.08 – 2.95 (m, J = 15.2, 7.8 Hz, 4H; H10, H6), 2.81 – 2.67 (m, 2H; H9), 2.36 (t, J = 7.1 Hz, 2H; H3), 1.92 – 1.79 (m, J = 12.4, 9.1, 6.2 Hz, 2H; H4), 1.77 – 1.64 (m, 2H; H5). **^{13}C NMR (101 MHz, CDCl$_3$)** δ 173.53 (C12), 153.87 (C2), 152.55 (C7), 113.75 (C11), 61.61(C13), 51.74 (C8), 47.58 (C1), 33.13 (C3), 28.84 (C5), 26.29 (C4), 24.24 (C6), 24.05 (C9), 21.12 (C10). **ATR-FTIR** (cm^{-1})**:** 2954, 1727, 1703, 1621, 1440, 1358, 1259, 1222, 1148, 1029, 911, 891, 860, 755, 714, 635. **HRMS (ESI+):** exact mass calculated for $[M]^+$ ($C_{13}H_{20}NO_4^+$) requires m/z 254.1387, found m/z 254.1389.

3-Ethyl-1-methoxy-5,6,7,8-tetrahydrooxazolo[3,4-a]pyridin-4-ium trifluoromethanesulfonate (2f)

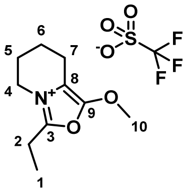

Prepared according to general procedure B as an yellow oil; 60 % yield (80 mg); **^{1}H NMR (400 MHz, CDCl$_3$)** δ 4.16 (t, J = 6.1 Hz, 2H; H4), 4.08 (s, 3H; H10), 3.04 (q, J = 7.5 Hz, 2H; H2), 2.69 (t, J = 6.5 Hz, 2H; H7), 2.09 – 1.99 (m, 2H; H5), 1.88 (dt, J = 5.3, 4.7 Hz, 2H; H6), 1.39 (t, J = 7.5 Hz, 3H; H1). **^{13}C NMR (101 MHz, CDCl$_3$)** δ 158.35 (C9), 154.33 (C3), 107.73 (C8), 62.37 (C10), 45.11 (C4), 20.90 (C5), 19.76 (C6), 17.84 (C2), 17.74 (C7), 8.33 (C1). **ATR-FTIR** (cm^{-1})**:** 2956, 1705, 1585, 1452, 1372, 1260, 1223, 1151, 1027, 956, 862, 795, 756, 697, 634. **HRMS (ESI+):** exact mass calculated for $[M]^+$ ($C_{10}H_{16}NO_2^+$) requires m/z 182.1176 , found m/z 182.1177.

3-(Cyclohexylmethyl)-1-methoxy-5,6,7,8-tetrahydrooxazolo[3,4-a]pyridin-4-ium trifluoromethanesulfonate (2g)

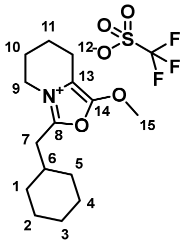

Prepared according to general procedure B as an yellow oil; 67 % yield (107 mg); **^{1}H NMR (400 MHz, CDCl$_3$)** δ 4.15 (t, J = 5.5 Hz, 2H; H9), 4.07 (s, 3H; H15), 2.88 (d, J = 7.0 Hz, 2H; H12), 2.69 (t, J = 6.3 Hz, 2H; H7), 2.07 – 2.00 (m, 2H; H10), 1.92 – 1.80 (m, 3H; H11, H6), 1.70 (d, J = 10.6 Hz, 4H; H1, H5), 1.35 – 0.97 (m, 6H; H2, H3, H4). **^{13}C NMR (101 MHz, CDCl$_3$)** δ 157.23 (C14), 154.40 (C8), 107.53 (C13), 62.34 (C15), 45.49 (C9), 35.69 (C6), 33.19 (C1, C5), 32.92 (C7), 25.69 (C3), 25.64 (C2, C4) , 20.96 (C10), 17.79 (C11), 17.70 (C12). **ATR-FTIR** (cm^{-1})**:** 2928, 2855, 1704, 1581, 1450, 1373, 1261, 1222, 1153, 1027, 958, 917, 862, 756, 731, 697, 635. **HRMS (ESI+):** exact mass calculated for $[M]^+$ ($C_{15}H_{24}NO_2^+$) requires m/z 250.1802 , found m/z 250.1809.

3-Butyl-1-methoxy-5,6,7,8-tetrahydrooxazolo[3,4-a]pyridin-4-ium trifluoromethanesulfonate (2h)

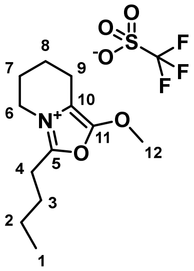

Prepared according to general procedure B as an yellow oil; 66.7 % yield (95.9 mg); **^{1}H NMR (400 MHz, CDCl$_3$)** δ 4.15 (t, J = 6.1 Hz, 2H; H6), 4.06 (s, 3H; H12), 2.97 (t, J = 7.8 Hz, 2H; H4), 2.67 (t, J = 6.5 Hz, 2H; H9), 2.06 – 1.97 (m, J = 11.0, 5.7 Hz, 2H; H7), 1.91 – 1.82 (m, 2H; H8), 1.80 – 1.71 (m, 2H; H3), 1.50 – 1.35 (m, 2H; H2), 0.93 (t, J = 7.3 Hz, 3H; H1). **^{13}C NMR (101 MHz, CDCl$_3$)** δ 157.77 (C11), 154.28 (C5), 107.60 (C10), 62.30 (C12), 45.17 (C6), 26.39 (C3), 25.57 (C4), 22.17 (C7), 20.89 (C8), 17.79 (C2) , 17.70 (C9), 13.44 (C1). **ATR-FTIR** (cm^{-1})**:** 2963, 2877, 1732, 1705, 1584, 1453, 1374, 1262, 1223, 1153, 1077, 1028, 959, 862, 824, 756, 736, 697, 634. **HRMS (ESI+):** exact mass calculated for $[M]^+$ ($C_{12}H_{20}NO_2^+$) requires m/z 210.1489, found m/z 210.1492.

3-(3-Bromopropyl)-1-methoxy-5,6,7,8-tetrahydrooxazolo[3,4-a]pyridin-4-ium trifluoromethanesulfonate (2i)

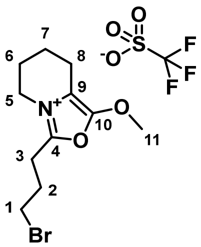

Prepared according to general procedure B as an yellow oil; 67 % yield (114 mg); **^{1}H NMR (400 MHz, CDCl$_3$)** δ 4.18 (t, J = 6.0 Hz, 2H; H5), 4.07 (s, 3H; H11), 3.66 (t, J = 6.0 Hz, 1H; H1), 3.52 (t, J = 6.1 Hz, 1H; H1), 3.19 (t, J = 7.3 Hz, 2H; H8), 2.69 (t, J = 6.4 Hz, 2H; H3), 2.46 – 2.27 (m, 2H; H2), 2.10 – 1.97 (m, J = 4.9 Hz, 2H; H6), 1.93 – 1.81 (m, J = 11.6, 5.9 Hz, 2H; H7). **^{13}C NMR (101 MHz, CDCl$_3$)** δ 156.30 (C10), 154.52 (C4), 107.89 (C9), 62.39 (C11), 45.40 (C5), 31.91 (C1), 27.42 (C2), 24.70 (C3), 20.87 (C6), 17.82 (C7), 17.72 (C8). **ATR-FTIR** (cm^{-1})**:** 2959, 1704, 1587, 1450, 1376, 1257, 1222, 1149, 1081, 1028, 956, 862, 823, 755, 735, 697. **HRMS (ESI+):** exact mass calculated for $[M]^+$ ($C_{11}H_{17}BrNO_2^+$) requires m/z 274.0437, found m/z 274.0439.

3-Isopentyl-1-methoxy-5,6,7,8-tetrahydrooxazolo[3,4-a]pyridin-4-ium trifluoromethanesulfonate (2j)

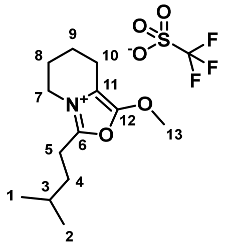

Prepared according to general procedure B as an yellow oil; 71 % yield (105 mg); **^{1}H NMR (400 MHz, $CDCl_3$)** δ 4.16 (t, *J* = 6.2 Hz, 2H; H7), 4.06 (s, 3H; H13), 2.97 (t, 2H; H10), 2.68 (t, *J* = 6.5 Hz, 2H; H5), 2.07 – 1.98 (m, *J* = 12.1, 6.2 Hz, 2H; H8), 1.93 – 1.81 (m, 2H; H9), 1.72 – 1.60 (m, 3H; H4, H3), 0.93 (d, *J* = 6.4 Hz, 6H; H1, H2). **^{13}C NMR (101 MHz, $CDCl_3$)** δ 157.97 (C12), 154.30 (C6), 107.70 (C11), 62.38 (C13), 45.17 (C7), 32.91 (C4), 27.72 (C3), 24.01 (C5), 21.98 (C1), 20.93 (C2), 17.82 (C8, C9), 17.73(C10). **ATR-FTIR** (cm^{-1})**:** 2960, 2875, 1706, 1585, 1453, 1373, 1259, 1222, 1152, 1078, 1029, 962, 862, 823, 756, 736, 698, 665, 635. **HRMS (ESI+):** exact mass calculated for $[M]^+$ ($C_{13}H_{22}NO_2^+$) requires *m/z* 224.1645, found *m/z* 224.1648.

Dimethyl 4-ethyl-2a-methoxy-7,8-dihydro-2aH,4H,6H-cyclobuta[d]pyrrolo[1,2-c]oxazole-1,2-dicarboxylate (6)

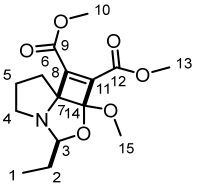

^{1}H NMR (400 MHz, $CDCl_3$) δ 4.29 (dd, *J* = 8.2, 6.3 Hz, 1H, H3), 3.83 (s, 3H; H15), 3.73 (s, 3H; H13), 3.71 (s, 3H; H10), 3.08 – 3.00 (m, 1H; H4), 2.77 – 2.64 (m, 2H; H4, H6), 2.00 – 1.66 (m, 5H; H6, H5, H2), 1.04 (t, *J* = 7.4 Hz, 3H; H1). **^{13}C NMR (101 MHz, $CDCl_3$)** δ 166.21 (C12), 163.08 (C9), 146.76 (C11), 135.34 (C8), 81.70 (C7), 70.71 (C3), 52.85 (C13), 52.48 (C10), 52.34 (C15), 48.92 (C4), 34.23 (C6), 25.82 (C5), 21.35 (C2), 12.49 (C1). **ATR-FTIR** (cm^{-1})**:** 2954, 2882, 1737, 1654, 1436, 1264, 1197, 1172, 1154, 1062, 1037, 853. **HRMS (ESI+):** exact mass calculated for $[M+H]^+$ ($C_{15}H_{22}NO_6^+$) requires m/z 312.1442, found m/z 312.1445.

VI. Spectra

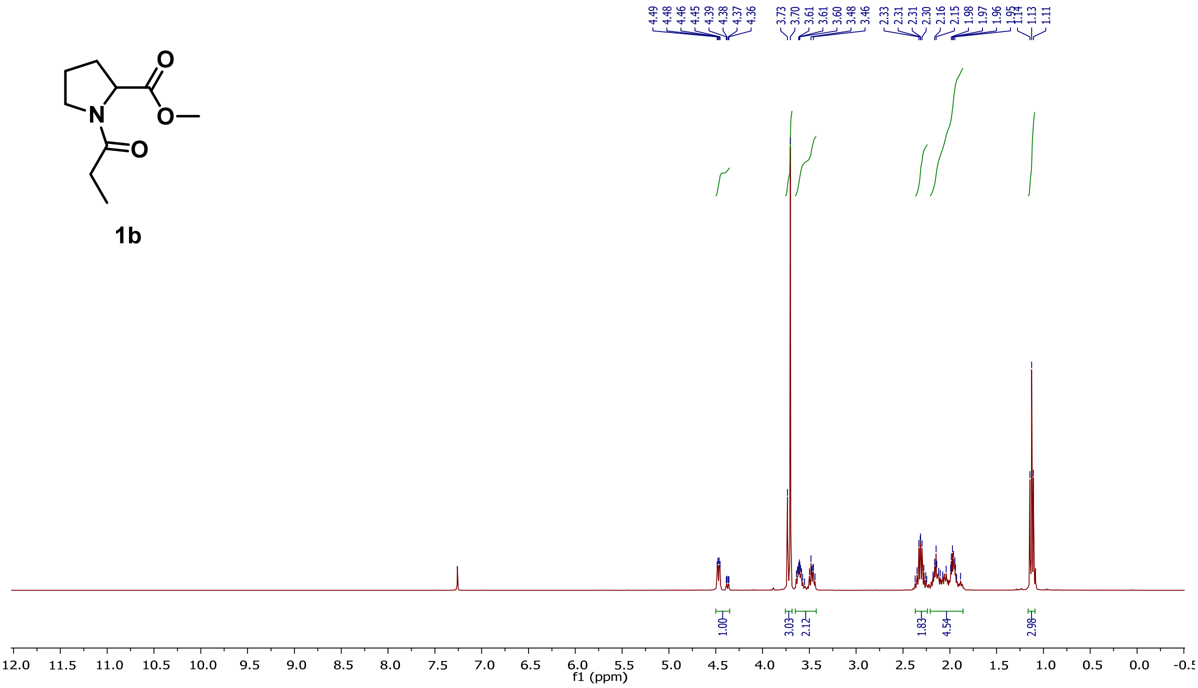

1b

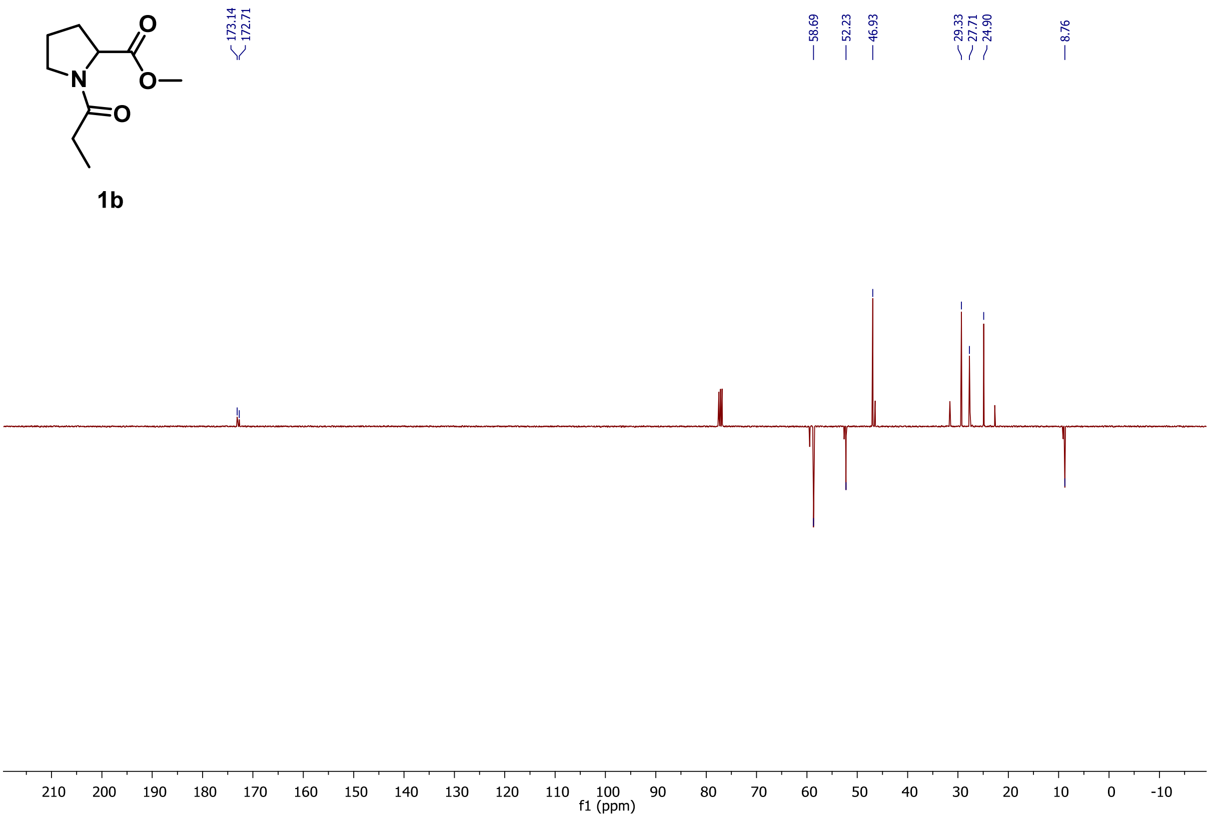

1b

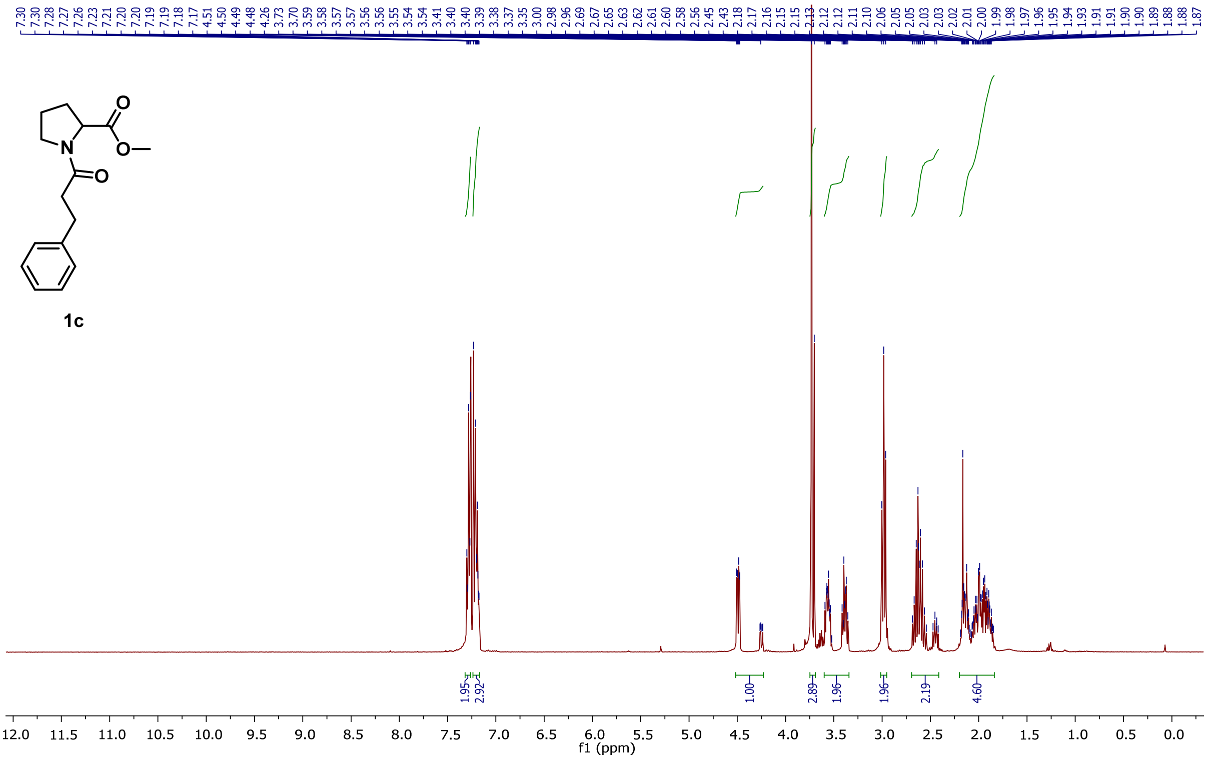

1c

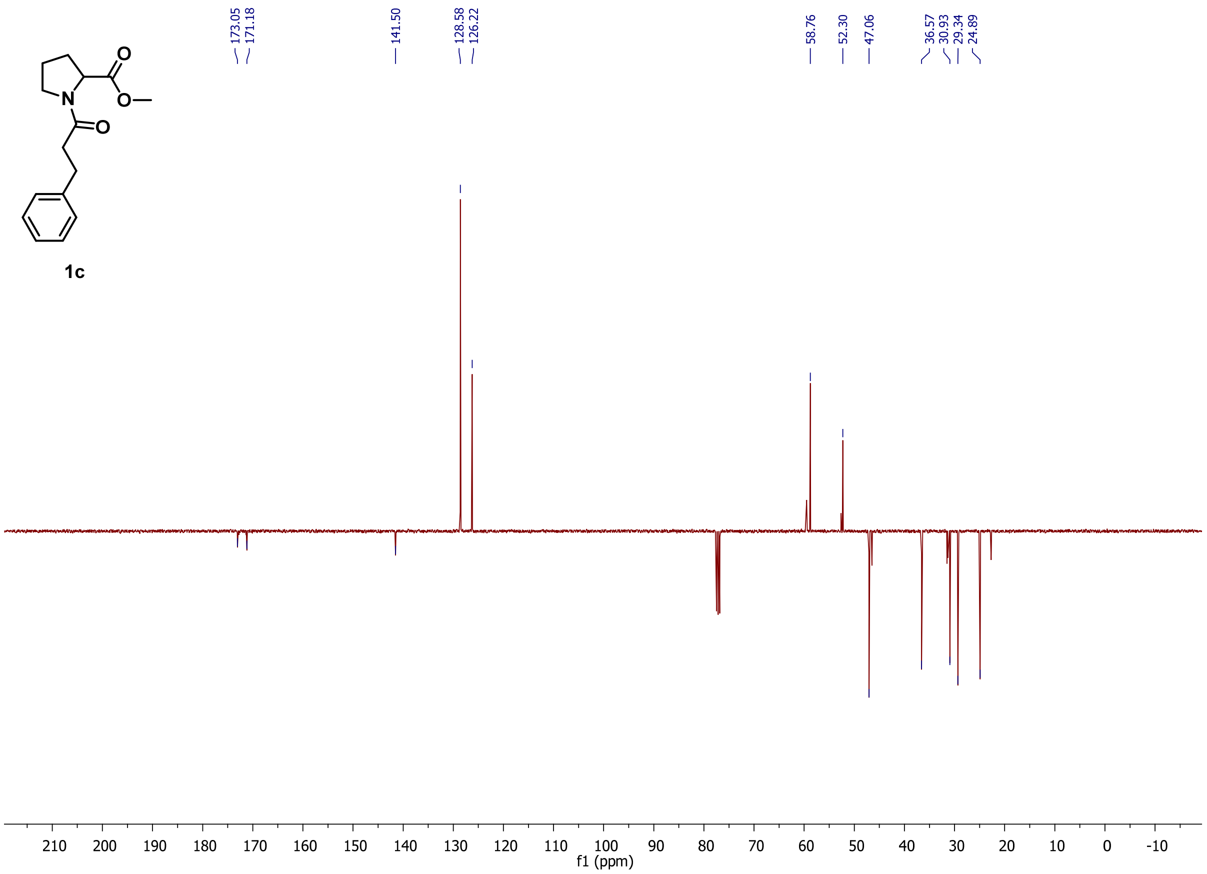

1c

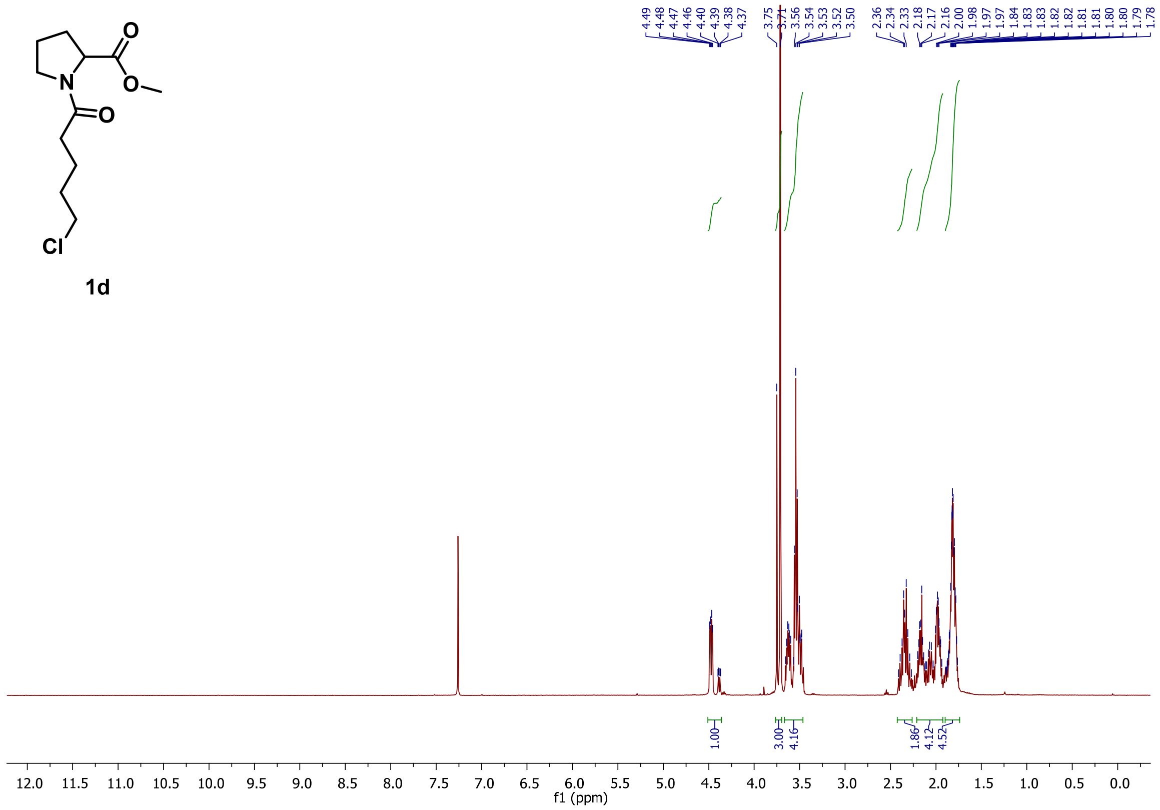
O
N
O
O
Cl
4.49
4.48
4.47
4.46
4.40
4.39
4.38
4.37
3.75
3.71
3.56
3.54
3.53
3.52
3.50
2.36
2.34
2.33
2.18
2.17
2.16
2.00
1.98
1.97
1.97
1.84
1.83
1.83
1.82
1.81
1.81
1.80
1.79
1.78
1.00
3.00
4.16
1.86
4.12
4.52
f1 (ppm)

1d

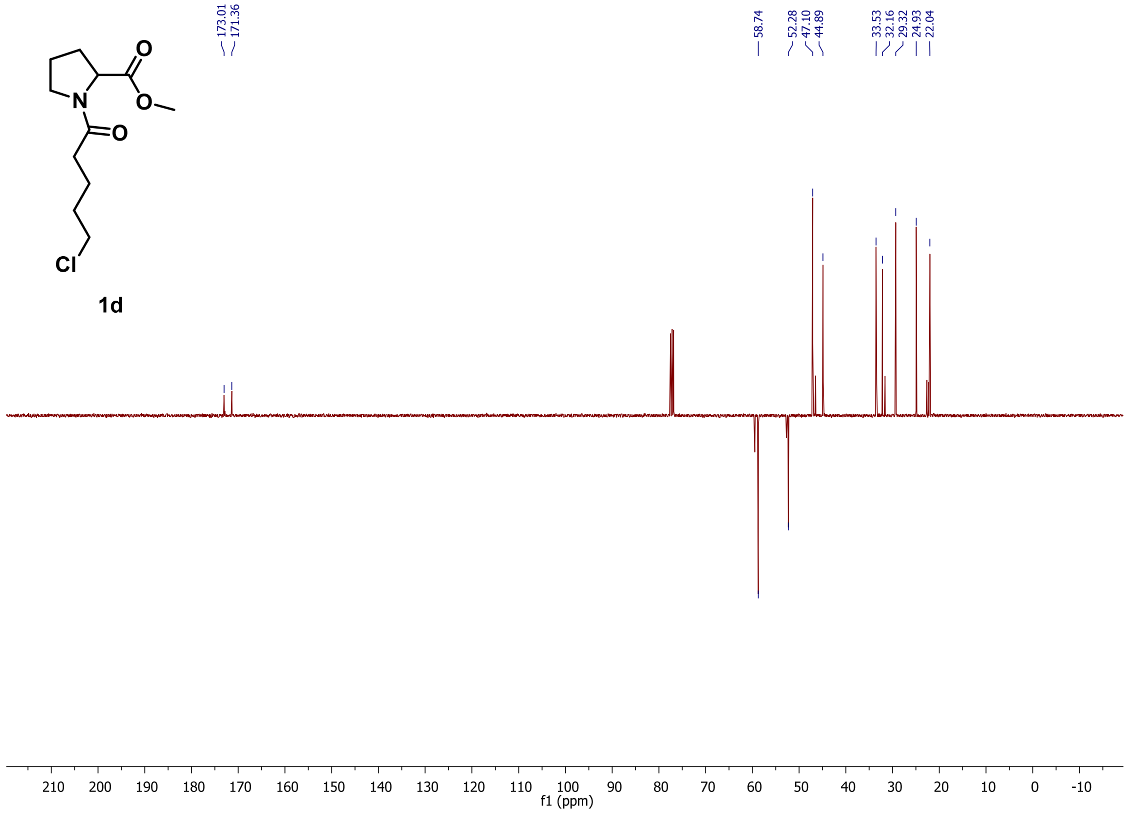
O
N
O
O
Cl
173.01
171.36
58.74
52.28
47.10
44.89
33.53
32.16
29.32
24.93
22.04
f1 (ppm)

1d

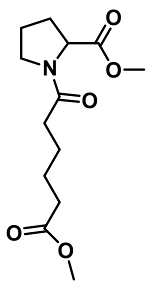

1e

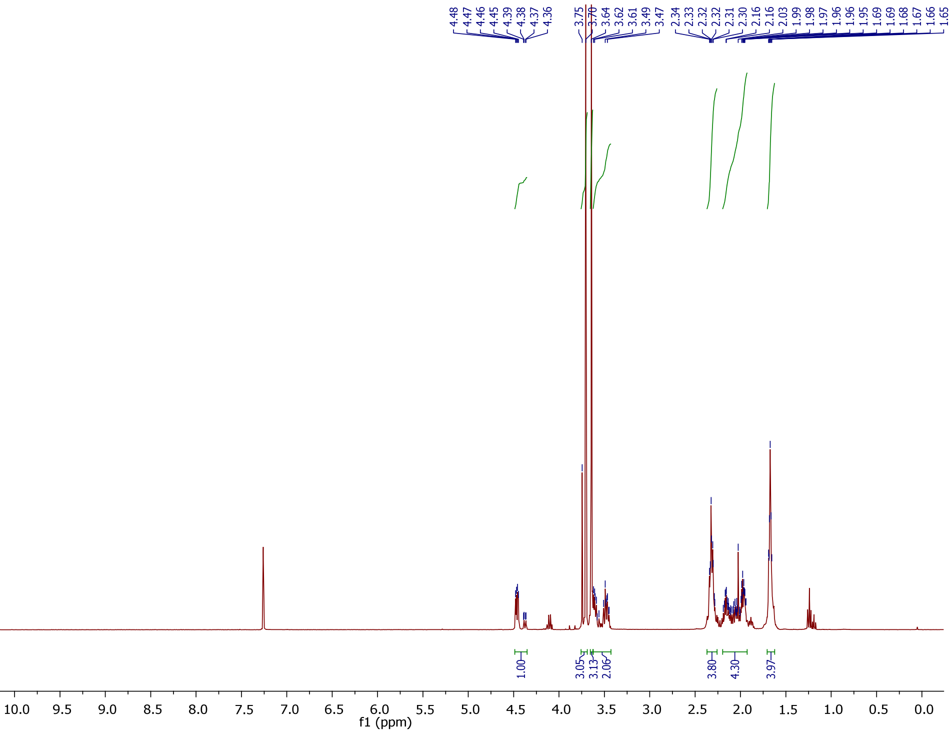
4.48 4.47 4.46 4.45 4.39 4.38 4.37 4.36 3.75 3.70 3.64 3.62 3.61 3.49 3.47 2.34 2.33 2.32 2.32 2.31 2.30 2.16 2.16 2.03 1.99 1.98 1.97 1.96 1.96 1.95 1.69 1.69 1.68 1.67 1.66 1.65
1.00 3.05 3.13 2.06 3.80 4.30 3.97
f1 (ppm)

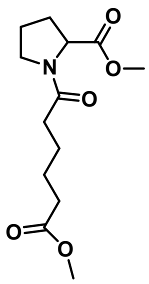

1e

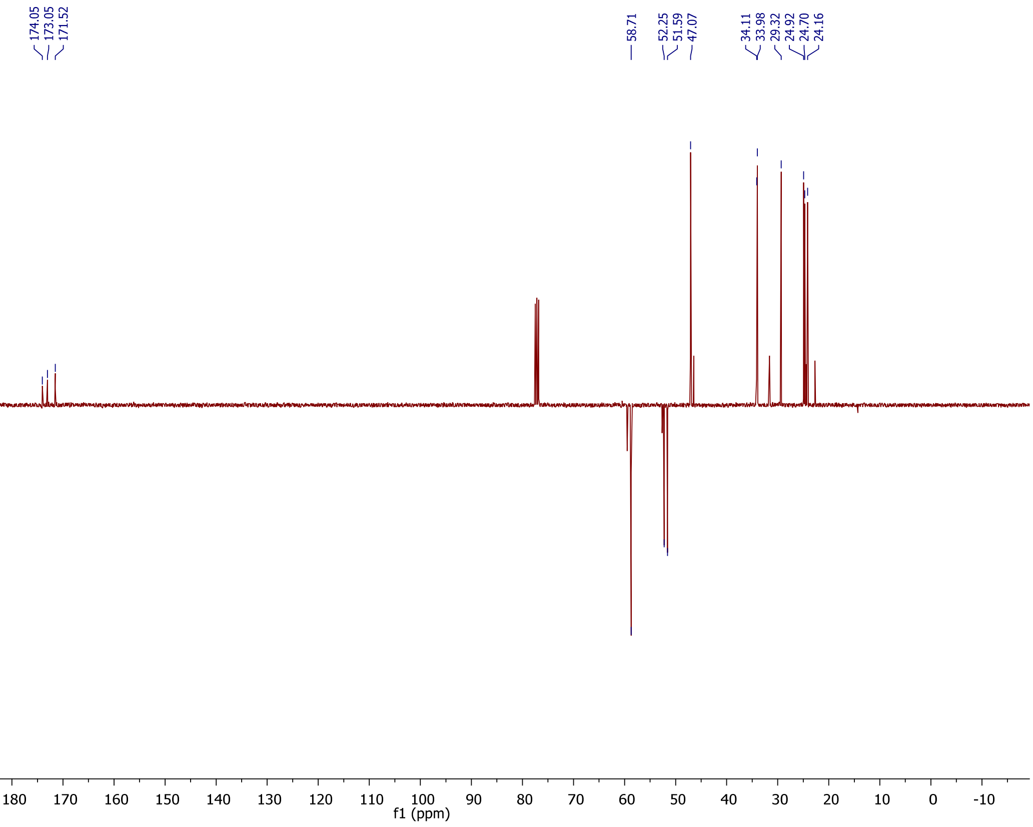
174.05 173.05 171.52
58.71
52.25 51.59 47.07
34.11 33.98 29.32 24.92 24.70 24.16
f1 (ppm)

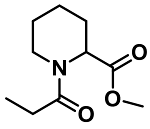
N
O
O
O

1f

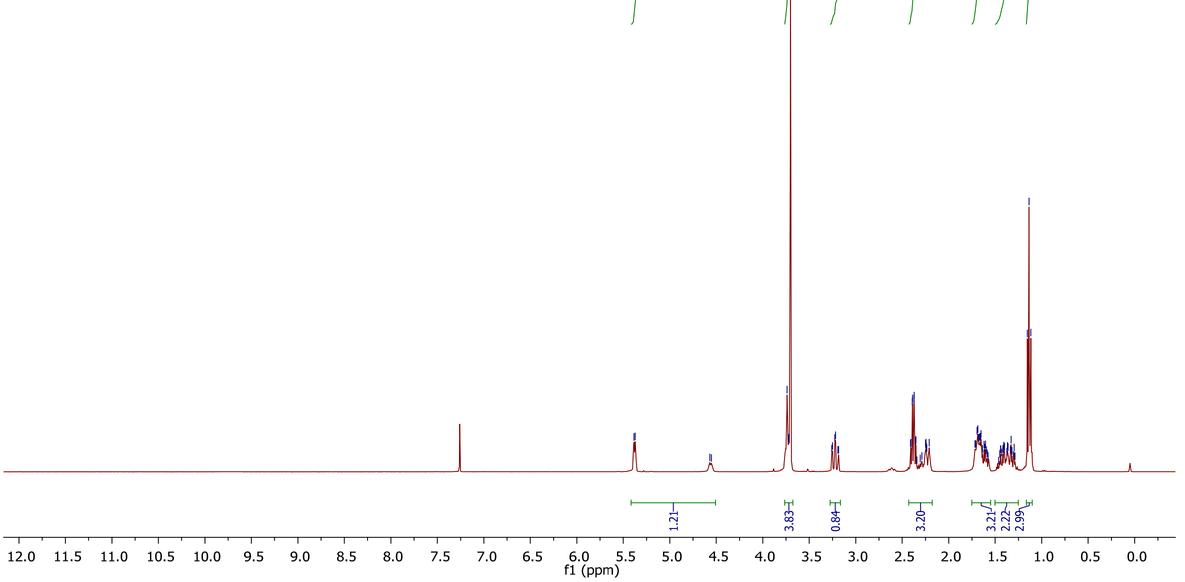
5.39
5.37
4.57
4.55
3.74
3.72
3.72
3.70
3.26
3.25
3.22
3.22
2.39
2.39
2.37
2.37
2.35
1.72
1.71
1.69
1.69
1.68
1.67
1.66
1.66
1.65
1.62
1.60
1.33
1.15
1.14
1.12
1.21
3.83
0.84
3.20
3.21
2.22
2.99
12.0 11.5 11.0 10.5 10.0 9.5 9.0 8.5 8.0 7.5 7.0 6.5 6.0 5.5 5.0 4.5 4.0 3.5 3.0 2.5 2.0 1.5 1.0 0.5 0.0
f1 (ppm)

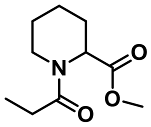
N
O
O
O

1f

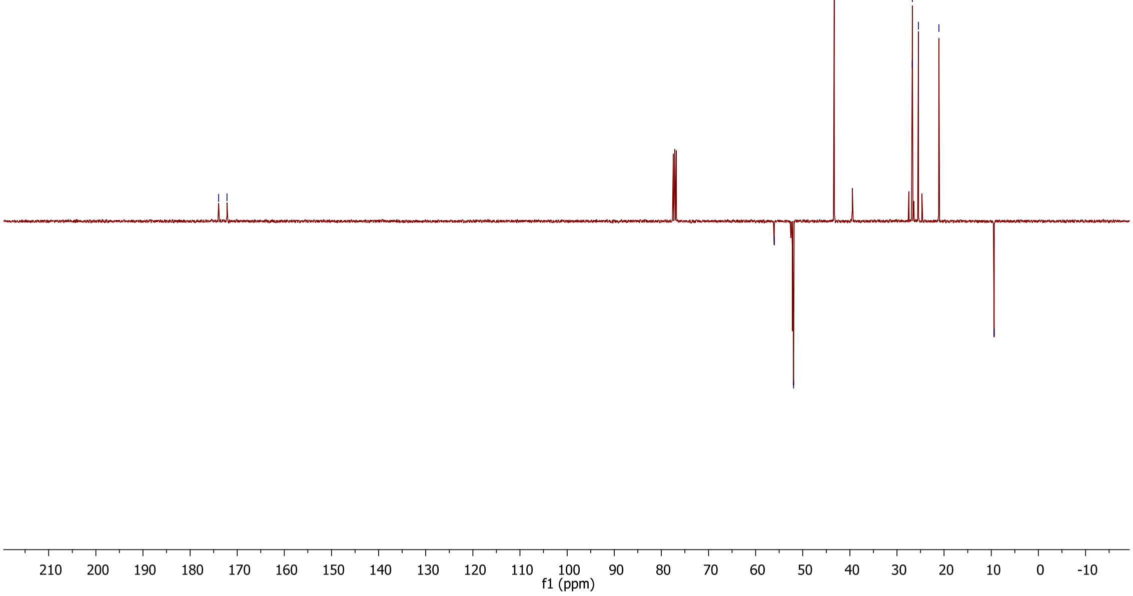
173.94
172.17
56.05
51.95
43.33
26.76
26.73
25.46
21.11
9.38
210 200 190 180 170 160 150 140 130 120 110 100 90 80 70 60 50 40 30 20 10 0 -10
f1 (ppm)

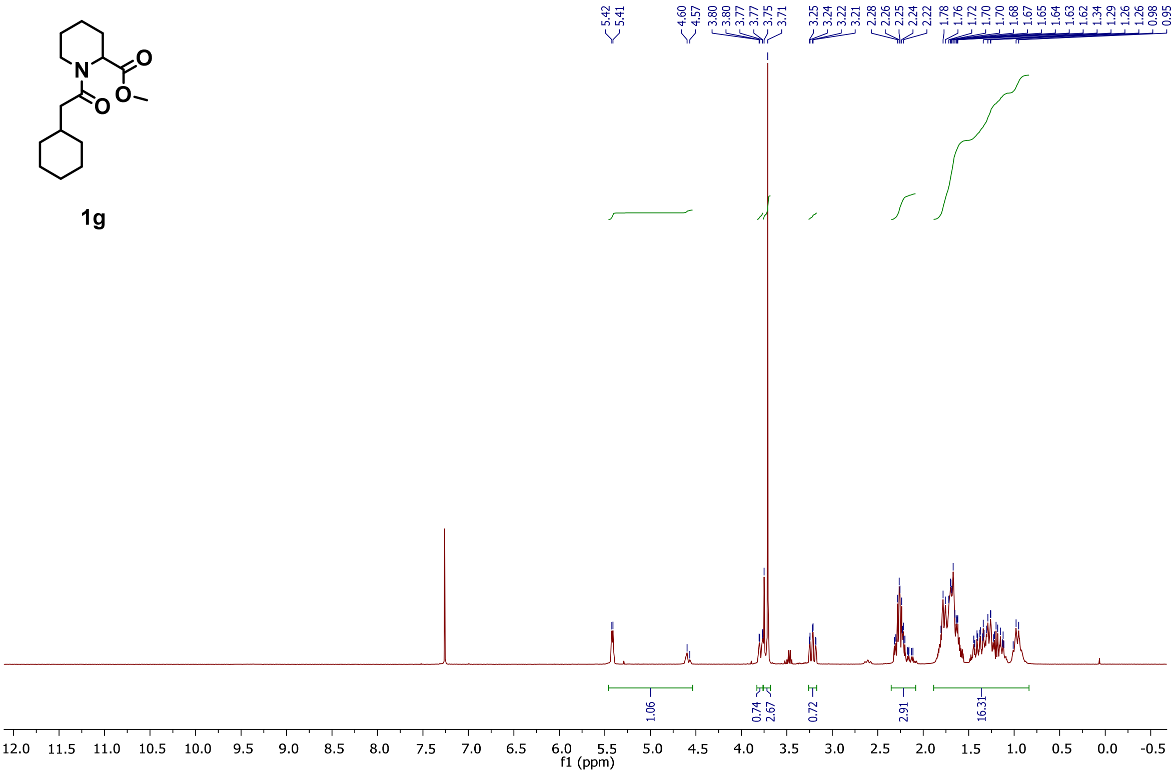

1g
5.42
5.41
4.60
4.57
3.80
3.80
3.77
3.77
3.75
3.71
3.25
3.24
3.22
3.21
2.28
2.26
2.25
2.24
2.22
1.78
1.76
1.72
1.70
1.70
1.68
1.67
1.65
1.64
1.63
1.62
1.34
1.29
1.26
1.26
0.98
0.95
1.06
0.74
2.67
0.72
2.91
16.31
12.0 11.5 11.0 10.5 10.0 9.5 9.0 8.5 8.0 7.5 7.0 6.5 6.0 5.5 5.0 4.5 4.0 3.5 3.0 2.5 2.0 1.5 1.0 0.5 0.0 -0.5
f1 (ppm)

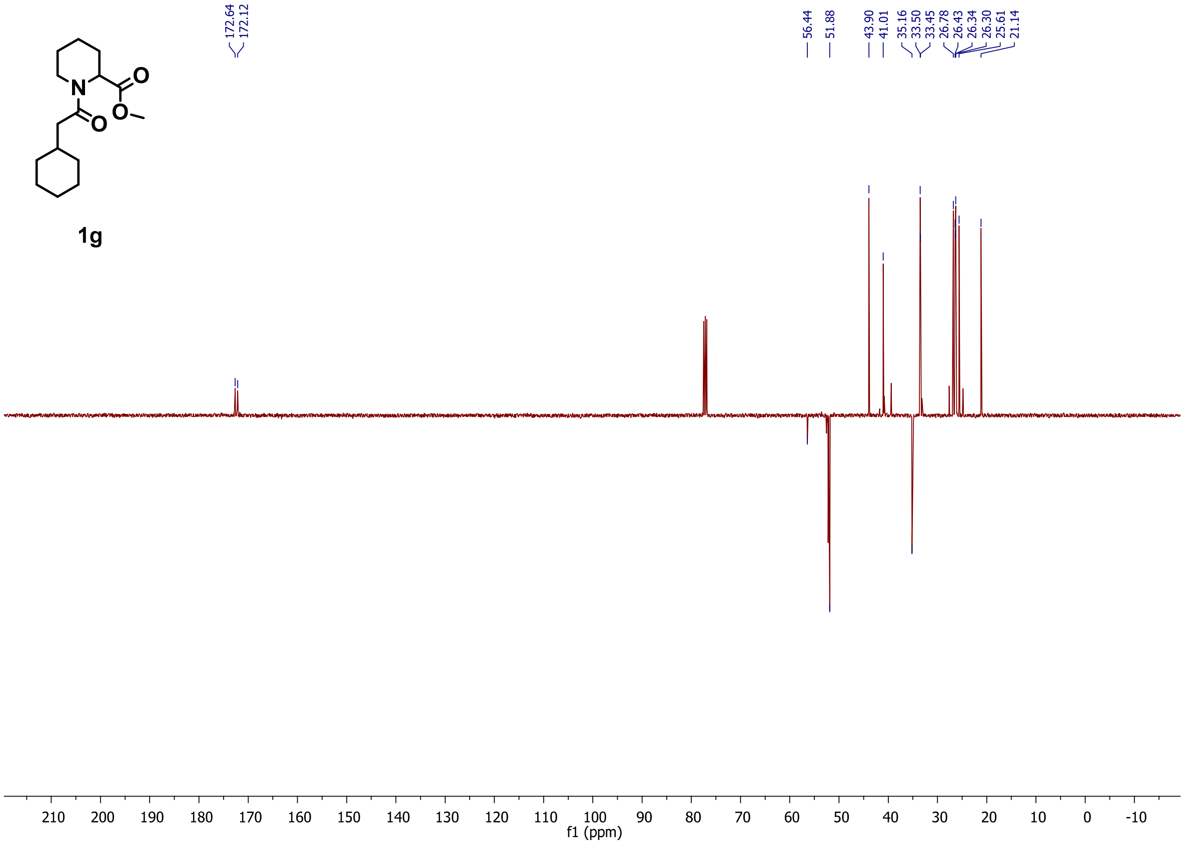

1g
172.64
172.12
56.44
51.88
43.90
41.01
35.16
33.50
33.45
26.78
26.43
26.34
26.30
25.61
21.14
210 200 190 180 170 160 150 140 130 120 110 100 90 80 70 60 50 40 30 20 10 0 -10
f1 (ppm)

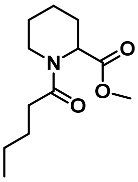
N
O
O
O

1h

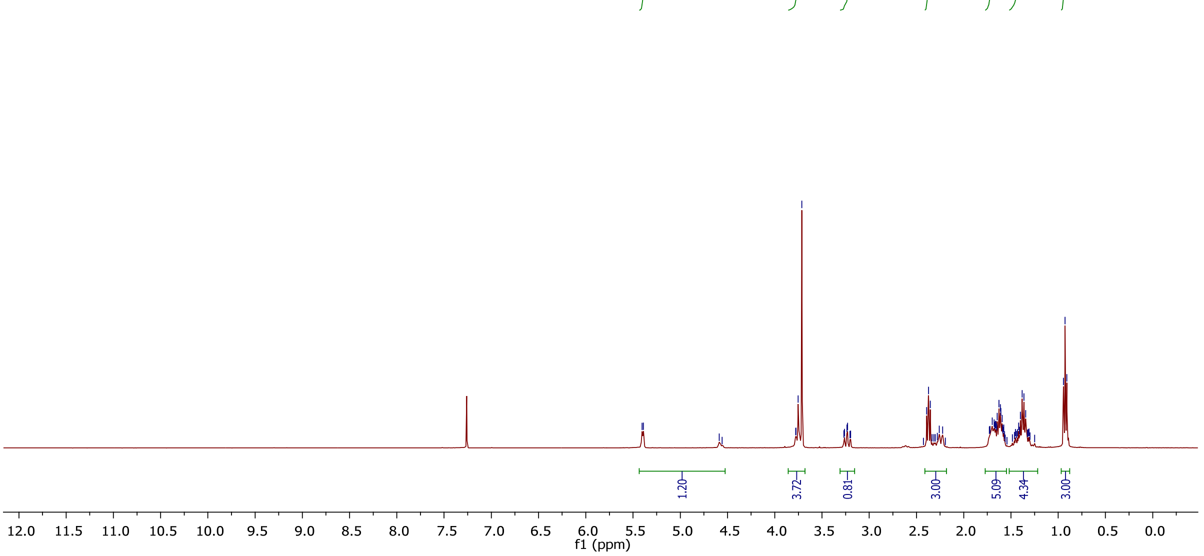
5.40
5.39
4.59
4.56
3.78
3.78
3.75
3.71
3.27
3.26
3.24
3.23
2.39
2.37
2.35
2.26
2.22
1.63
1.61
1.61
1.38
1.36
0.94
0.93
0.91
1.20
3.72
0.81
3.00
5.09
4.34
3.00
f1 (ppm)

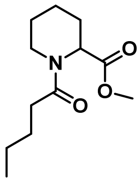
N
O
O
O

1h

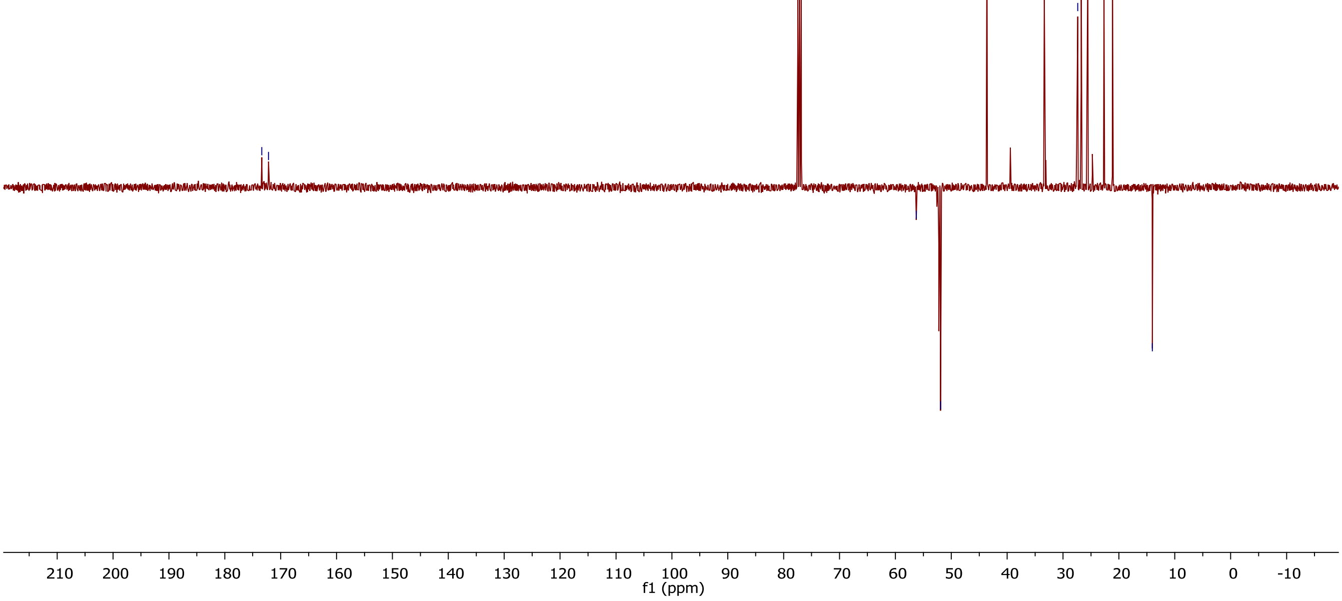
173.39
172.18
56.26
51.91
43.60
33.36
27.38
26.77
25.57
22.66
21.15
14.02
f1 (ppm)

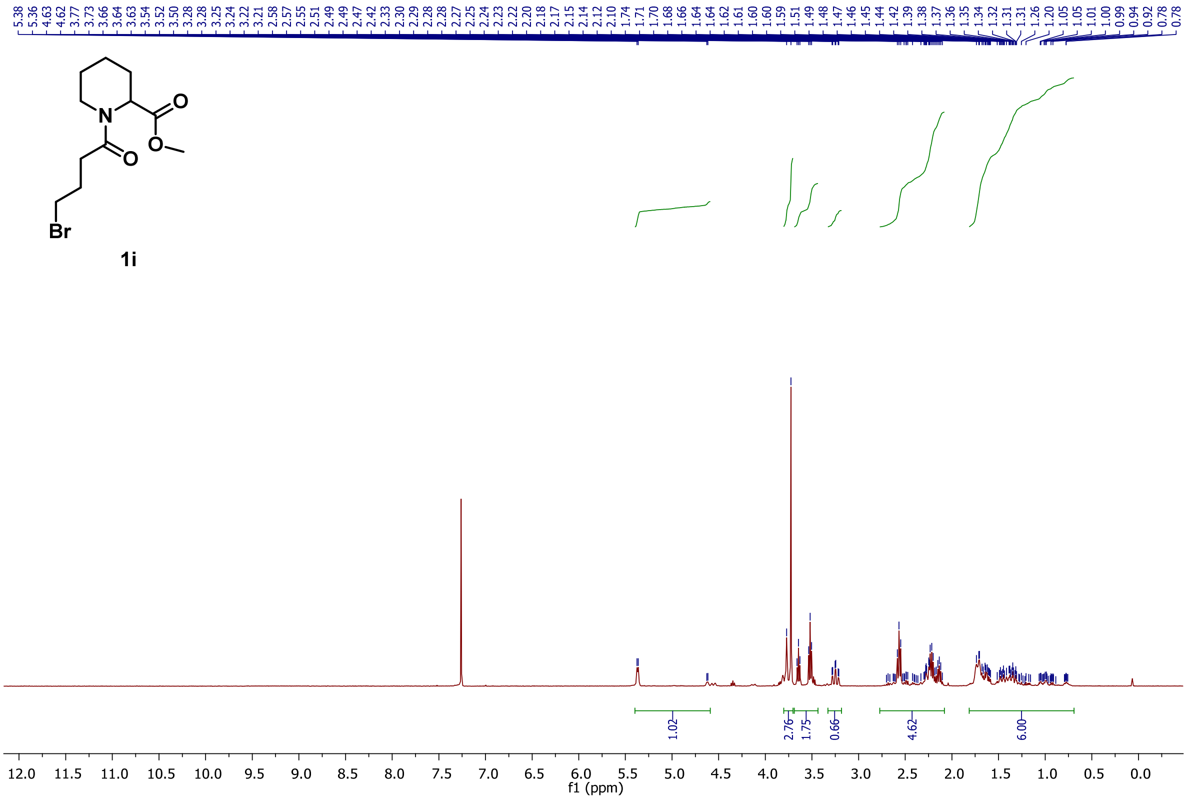
Br
1.02
2.76
1.75
0.66
4.62
6.00
f1 (ppm)

1i

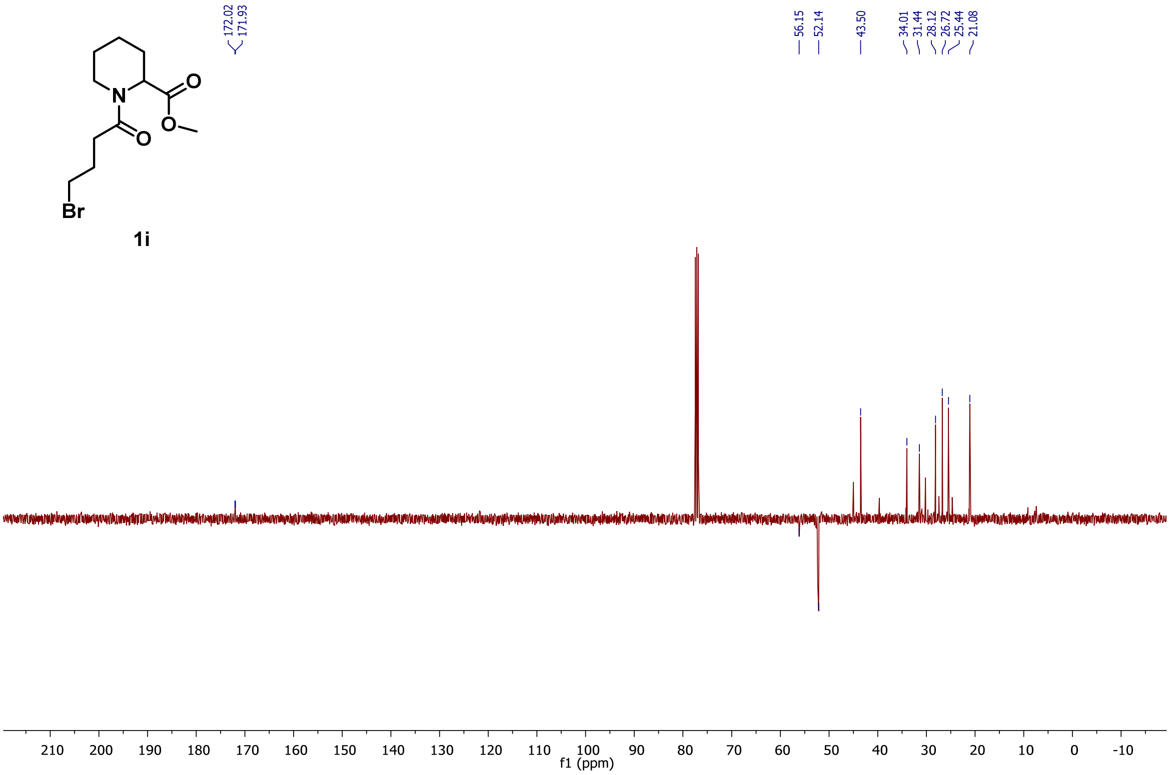
172.02
171.93
56.15
52.14
43.50
34.01
31.44
28.12
26.72
25.44
21.08
Br
f1 (ppm)

1i

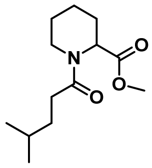

1j

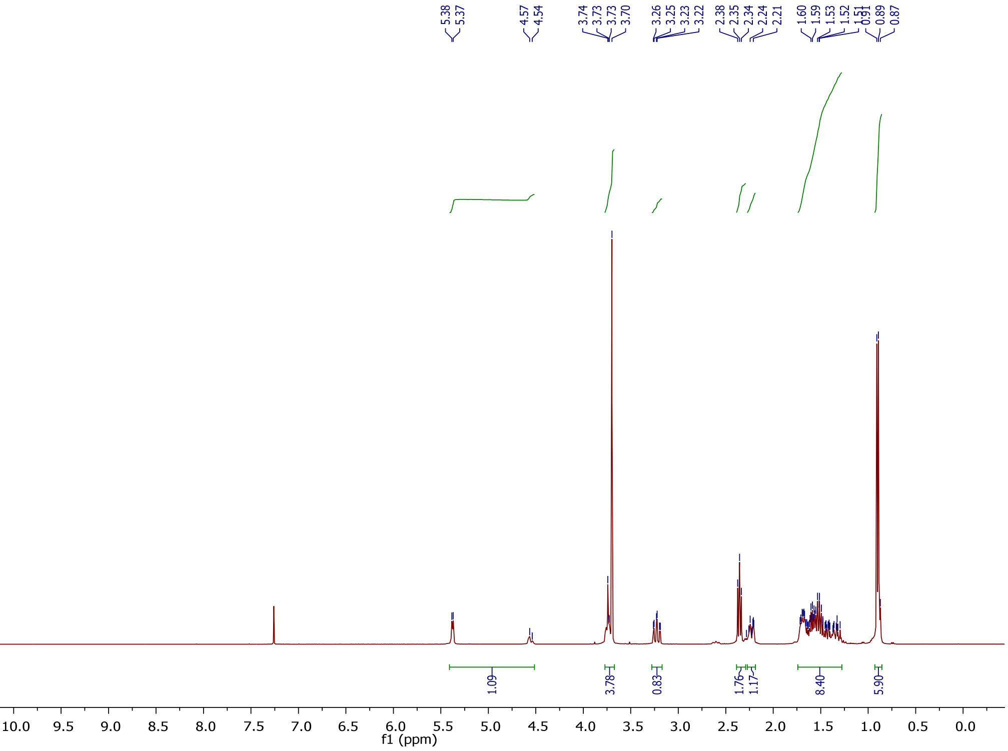
5.38
5.37
4.57
4.54
3.74
3.73
3.73
3.70
3.26
3.25
3.23
3.22
2.38
2.35
2.34
2.24
2.21
1.60
1.59
1.53
1.52
1.51
0.91
0.89
0.87
1.09
3.78
0.83
1.76
1.17
8.40
5.90
12.0 11.5 11.0 10.5 10.0 9.5 9.0 8.5 8.0 7.5 7.0 6.5 6.0 5.5 5.0 4.5 4.0 3.5 3.0 2.5 2.0 1.5 1.0 0.5 0.0
f1 (ppm)

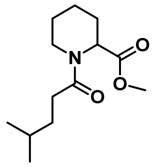

1j

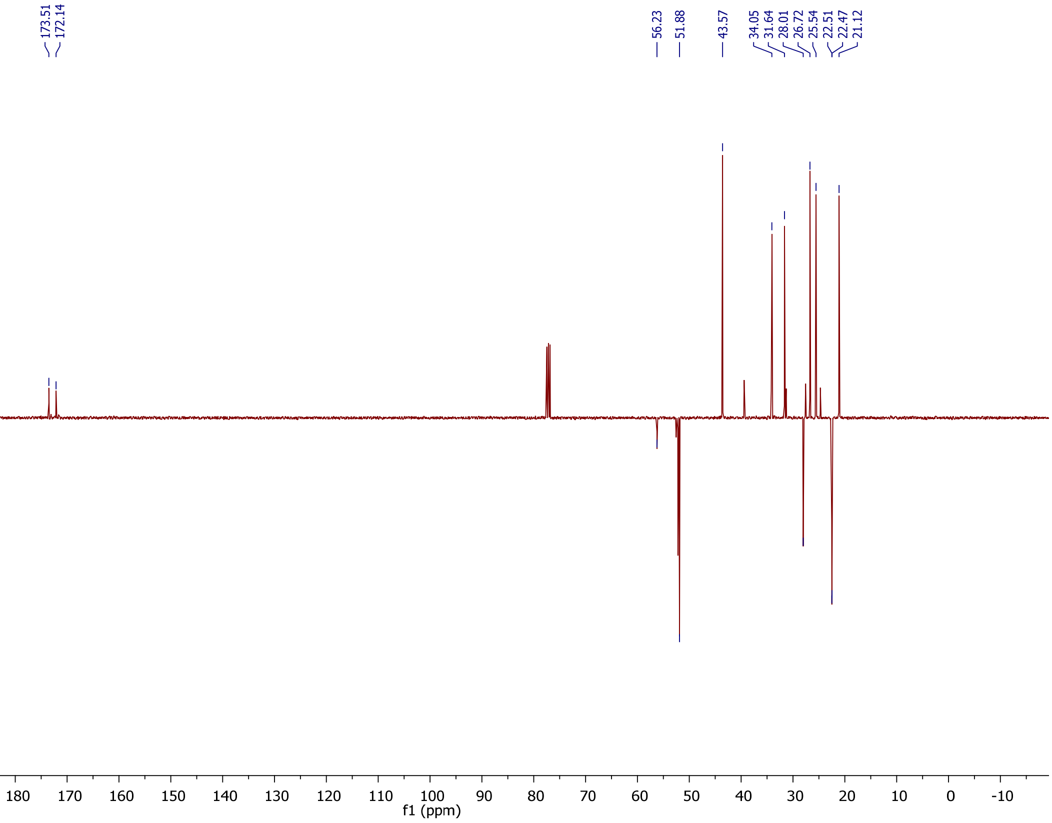
173.51
172.14
56.23
51.88
43.57
34.05
31.64
28.01
26.72
25.54
22.51
22.47
21.12
210 200 190 180 170 160 150 140 130 120 110 100 90 80 70 60 50 40 30 20 10 0 -10
f1 (ppm)

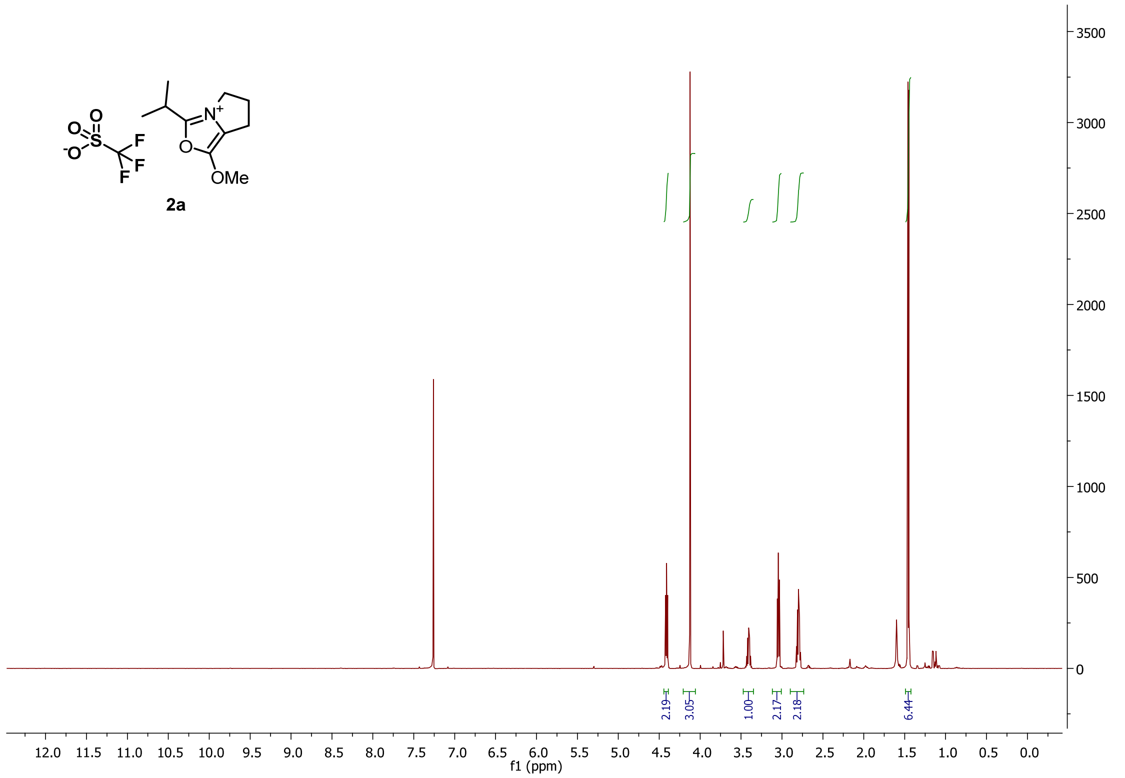
2a
OMe
2.19
3.05
1.00
2.17
2.18
6.44
f1 (ppm)

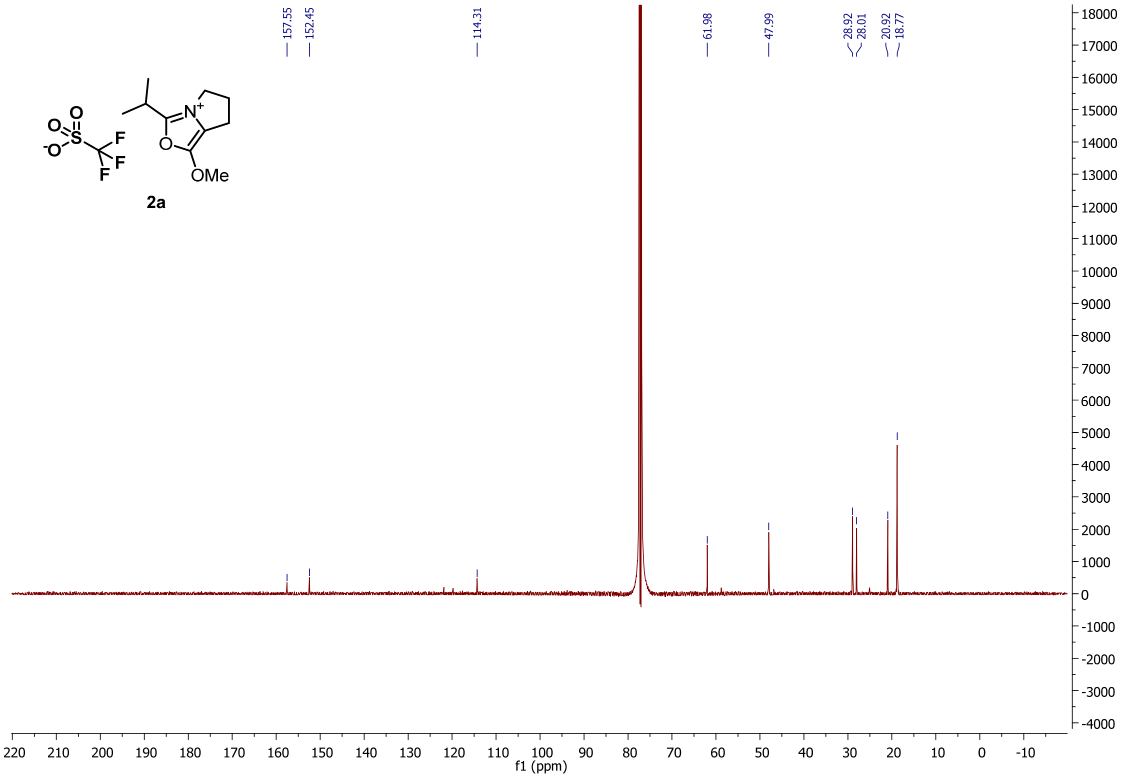
157.55
152.45
114.31
61.98
47.99
28.92
28.01
20.92
18.77
2a
OMe
f1 (ppm)

E. Spinozzi, A. Bauer, N. Maulide

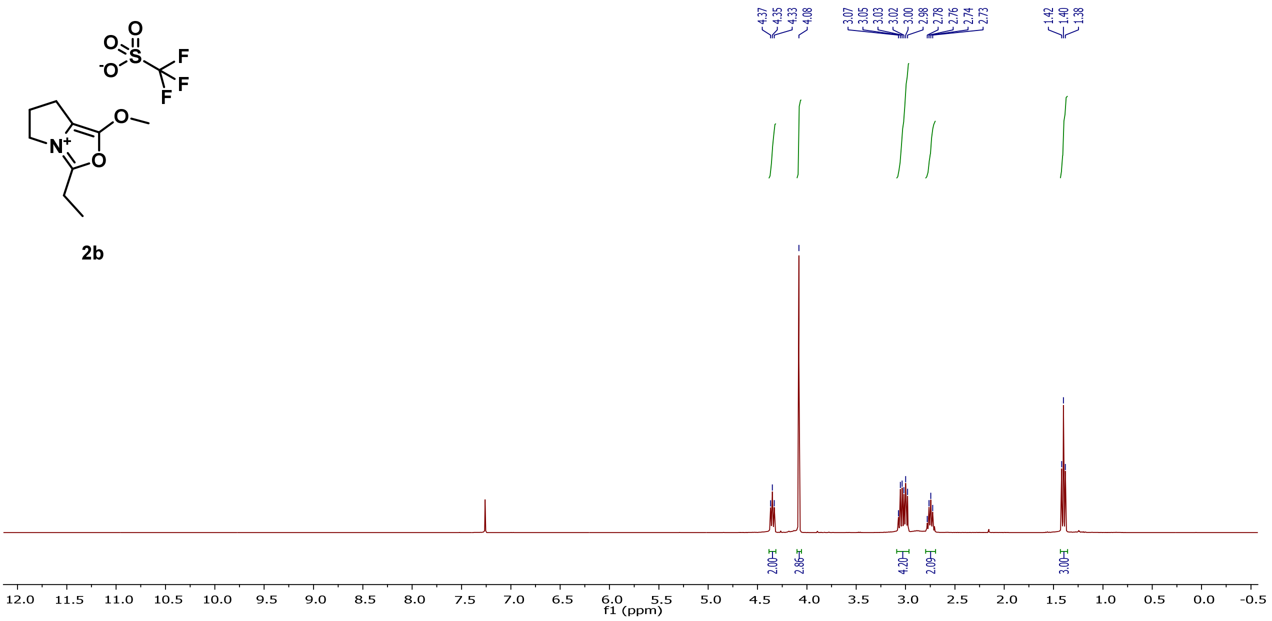

2b

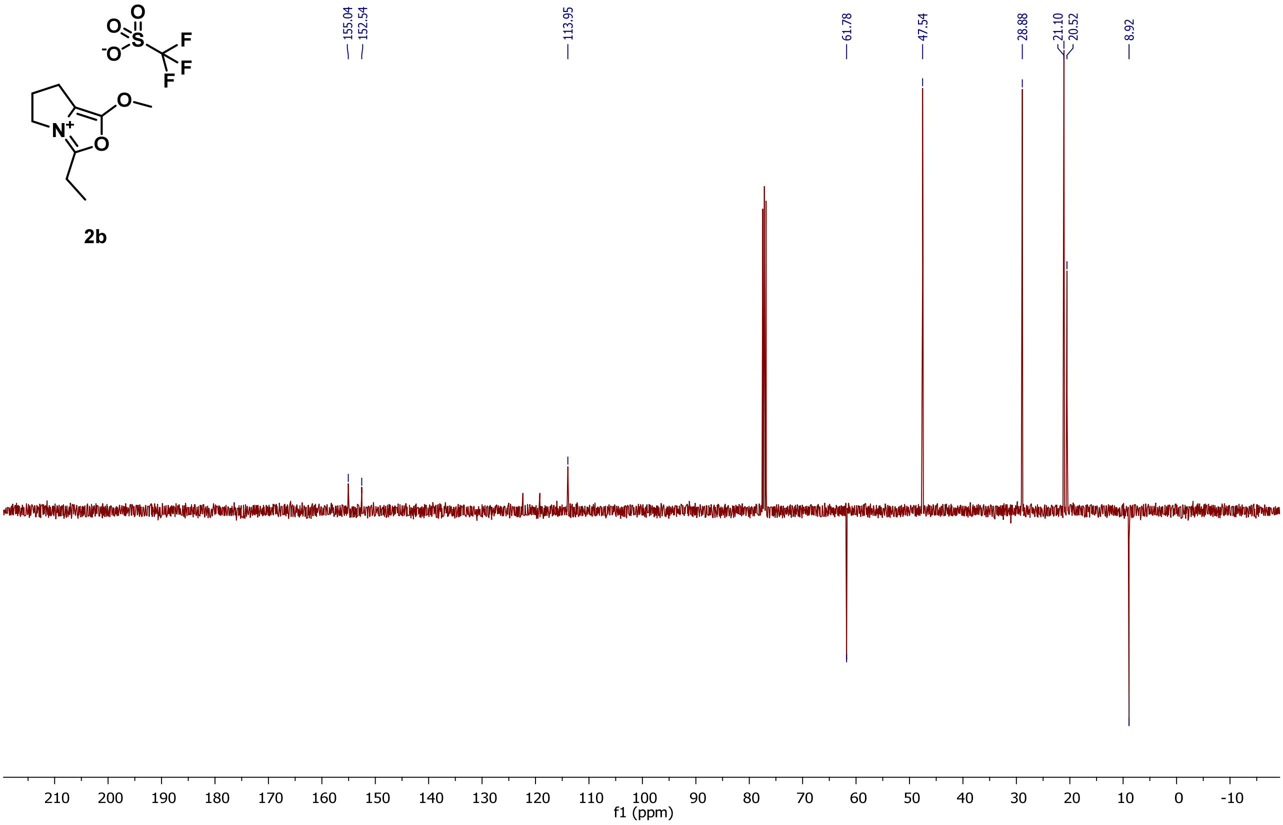

2b

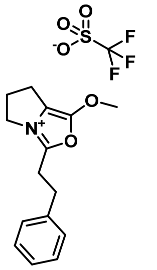

2c

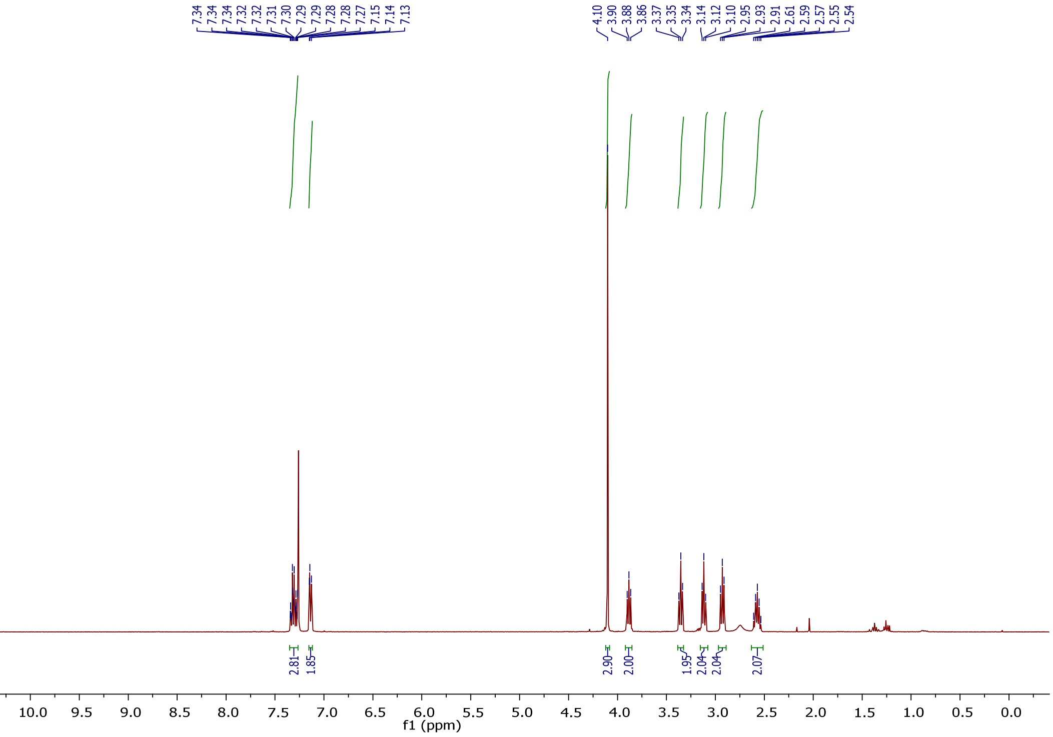

7.34 7.34 7.34 7.32 7.32 7.31 7.30 7.29 7.29 7.29 7.28 7.28 7.27 7.15 7.14 7.13
4.10 3.90 3.88 3.86 3.37 3.35 3.34 3.14 3.12 3.10 2.95 2.93 2.91 2.61 2.59 2.57 2.55 2.54
2.81 1.85 2.90 2.00 1.95 2.04 2.04 2.07
12.0 11.5 11.0 10.5 10.0 9.5 9.0 8.5 8.0 7.5 7.0 6.5 6.0 5.5 5.0 4.5 4.0 3.5 3.0 2.5 2.0 1.5 1.0 0.5 0.0
f1 (ppm)

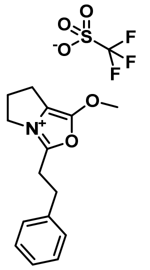

2c

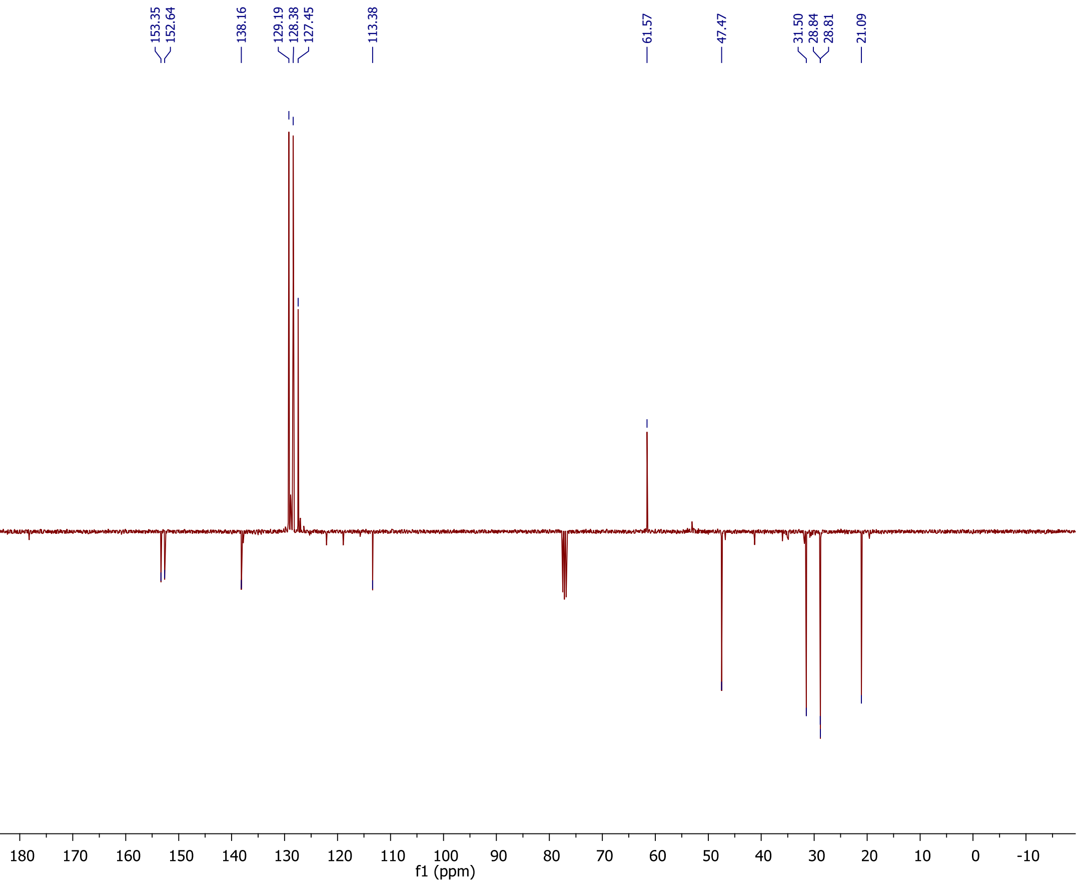

153.35 152.64 138.16 129.19 128.38 127.45 113.38 61.57 47.47 31.50 28.84 28.81 21.09
210 200 190 180 170 160 150 140 130 120 110 100 90 80 70 60 50 40 30 20 10 0 -10
f1 (ppm)

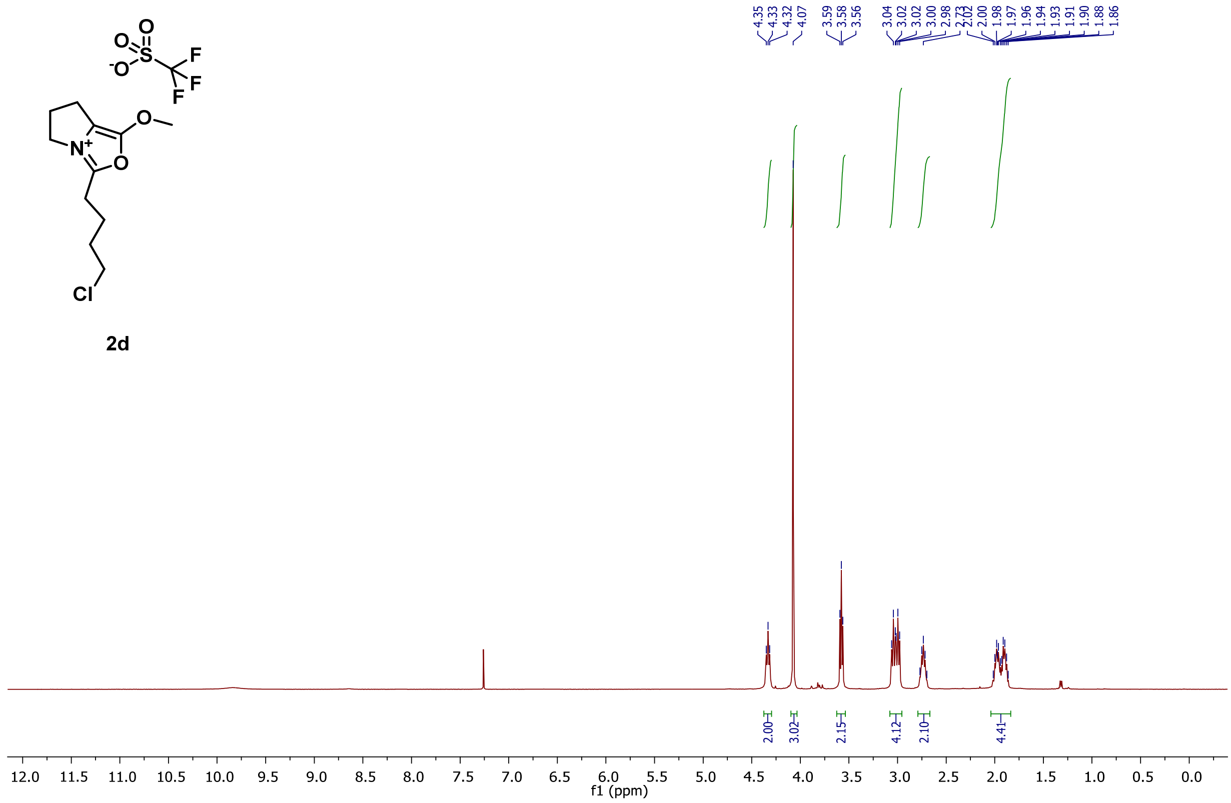
4.35
4.33
4.32
4.07
3.59
3.58
3.56
3.04
3.02
3.02
3.00
2.98
2.73
2.02
2.00
1.98
1.97
1.96
1.94
1.93
1.91
1.90
1.88
1.86
2.00
3.02
2.15
4.12
2.10
4.41
f1 (ppm)
2d

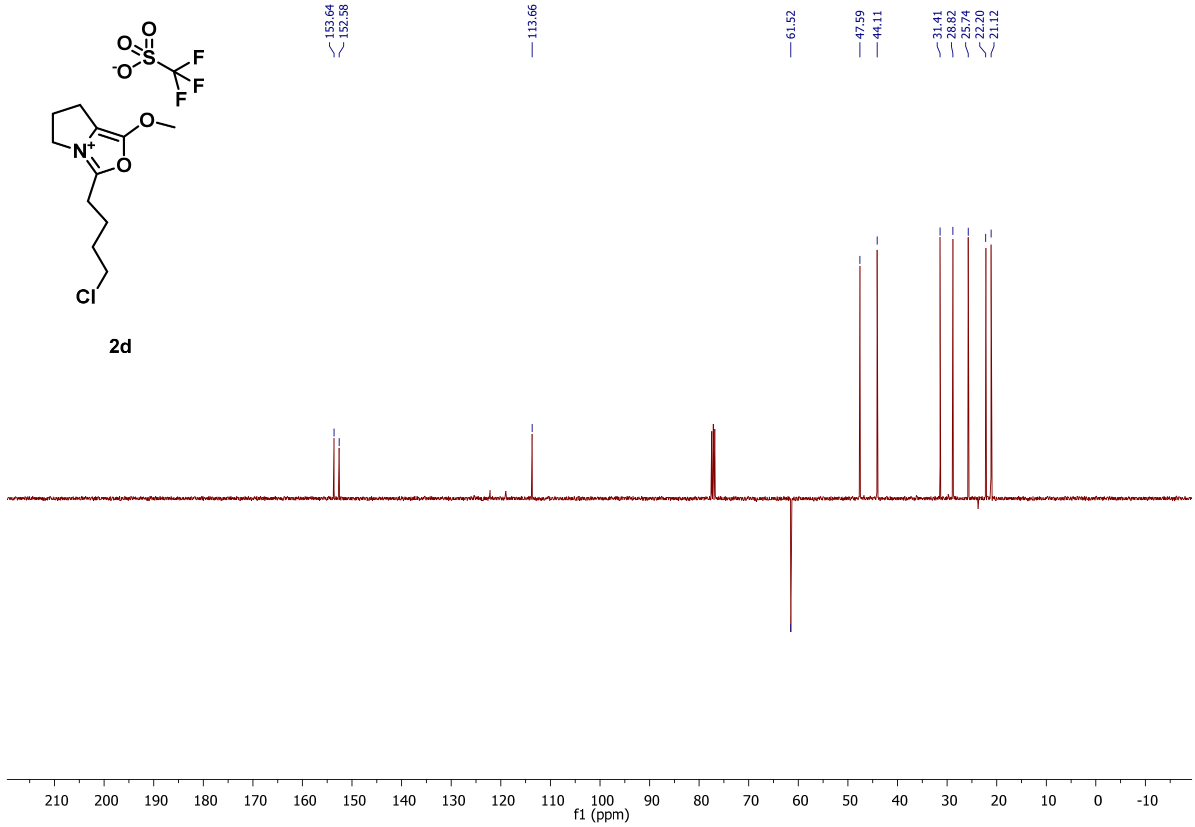
153.64
152.58
113.66
61.52
47.59
44.11
31.41
28.82
25.74
22.20
21.12
f1 (ppm)
2d

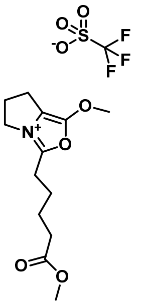
O
O
S
-O
F
F
F
N+
O
O
O
O

2e

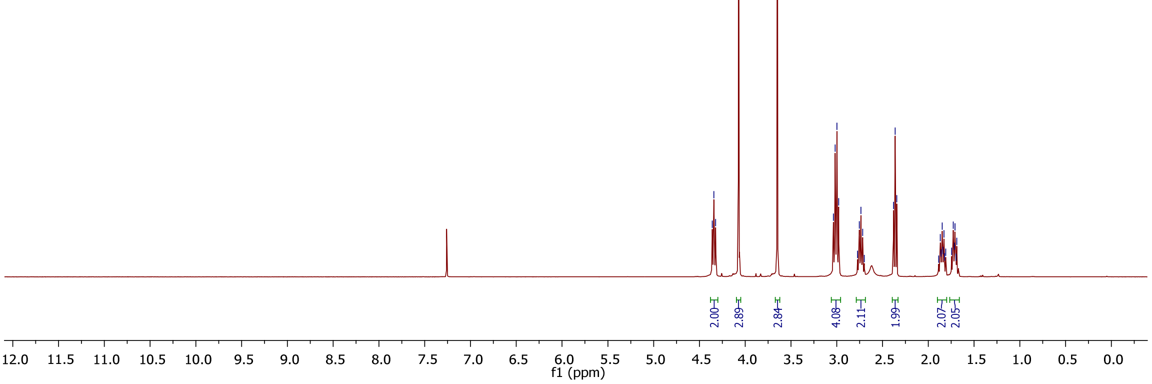
4.36
4.34
4.32
4.07
3.65
3.04
3.02
3.00
2.98
2.75
2.74
2.36
2.34
[illegible]
1.87
1.86
1.85
1.84
1.83
1.82
1.81
1.74
1.73
1.71
1.70
1.69
f1 (ppm)

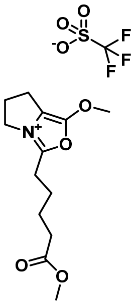
O
O
S
-O
F
F
F
N+
O
O
O
O

2e

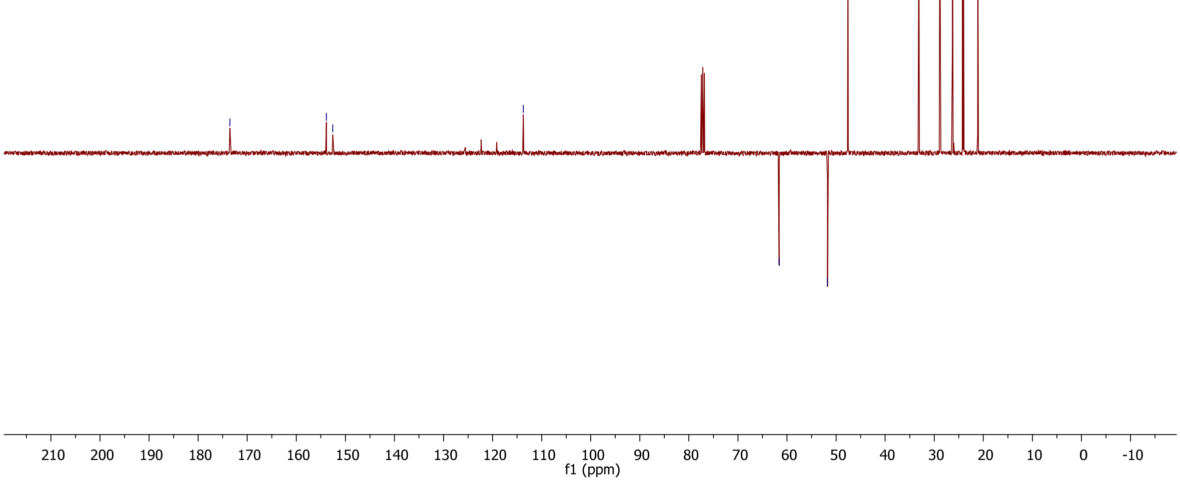
173.53
153.87
152.55
113.75
61.61
51.74
47.58
33.13
28.84
26.29
24.24
24.05
21.12
f1 (ppm)

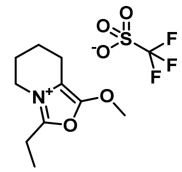

O
O
S
-O
F
F
F
N+
O
O

2f

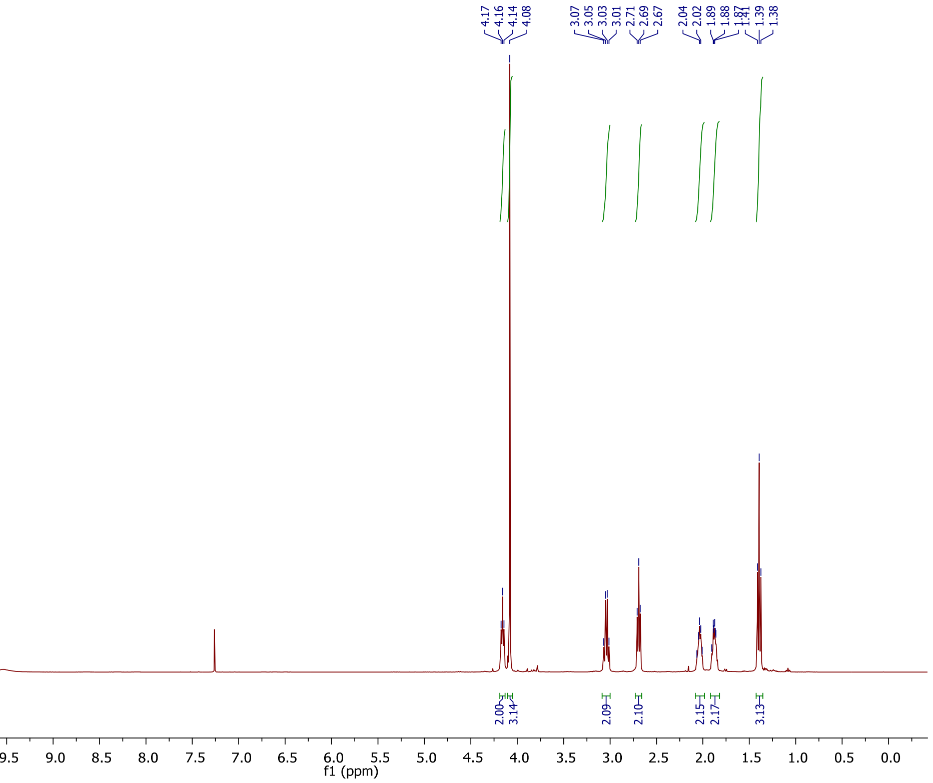

4.17
4.16
4.14
4.08
3.07
3.05
3.03
3.01
2.71
2.69
2.67
2.04
2.02
1.89
1.88
1.41
1.39
1.38
2.00
3.14
2.09
2.10
2.15
2.17
3.13
12.0 11.5 11.0 10.5 10.0 9.5 9.0 8.5 8.0 7.5 7.0 6.5 6.0 5.5 5.0 4.5 4.0 3.5 3.0 2.5 2.0 1.5 1.0 0.5 0.0
f1 (ppm)

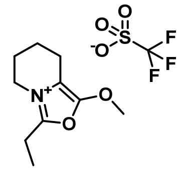

O
O
S
-O
F
F
F
N+
O
O

2f

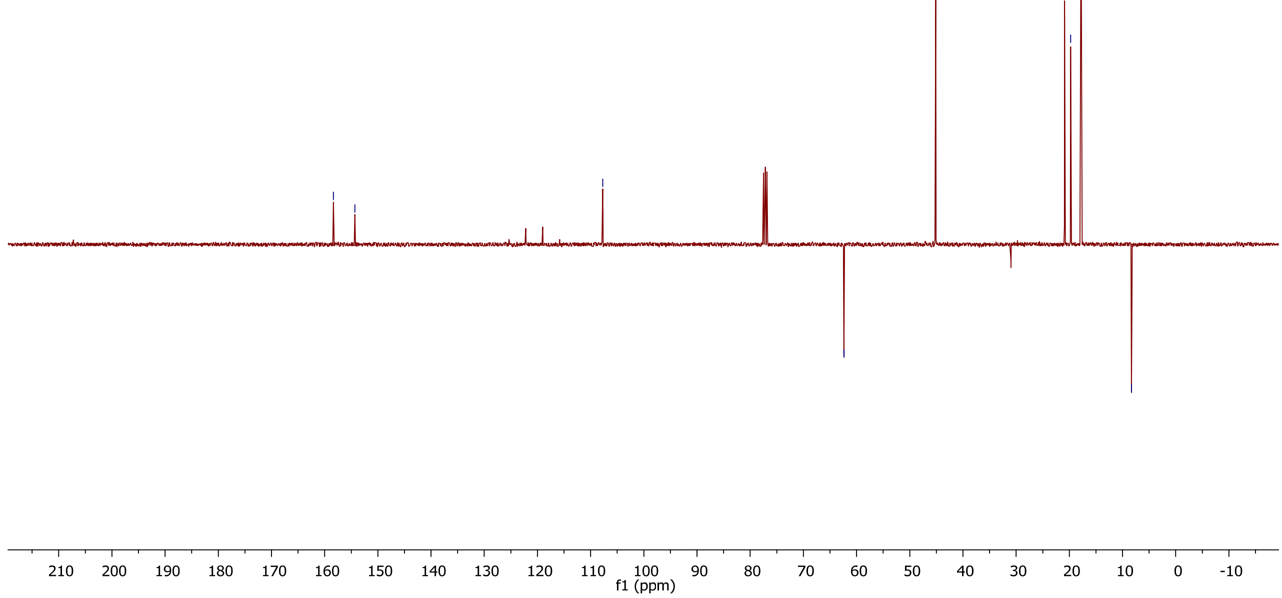

158.35
154.33
107.73
62.37
45.11
20.90
19.76
17.84
17.74
8.33
210 200 190 180 170 160 150 140 130 120 110 100 90 80 70 60 50 40 30 20 10 0 -10
f1 (ppm)

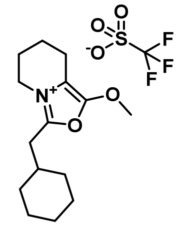
O
O
S
-O
F
F
F
N+
O
O

2g

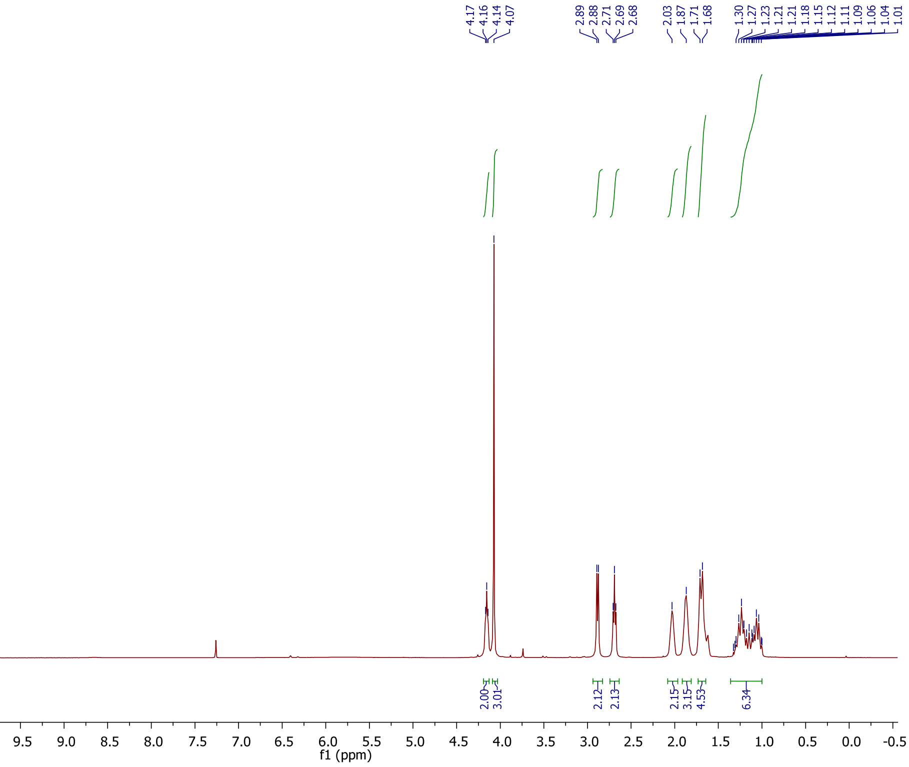
4.17
4.16
4.14
4.07
2.89
2.88
2.71
2.69
2.68
2.03
1.87
1.71
1.68
1.30
1.27
1.23
1.21
1.21
1.18
1.15
1.12
1.11
1.09
1.06
1.04
1.01
2.00
3.01
2.12
2.13
2.15
3.15
4.53
6.34
12.0 11.5 11.0 10.5 10.0 9.5 9.0 8.5 8.0 7.5 7.0 6.5 6.0 5.5 5.0 4.5 4.0 3.5 3.0 2.5 2.0 1.5 1.0 0.5 0.0 -0.5
f1 (ppm)

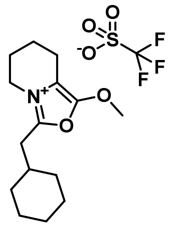
O
O
S
-O
F
F
F
N+
O
O

2g

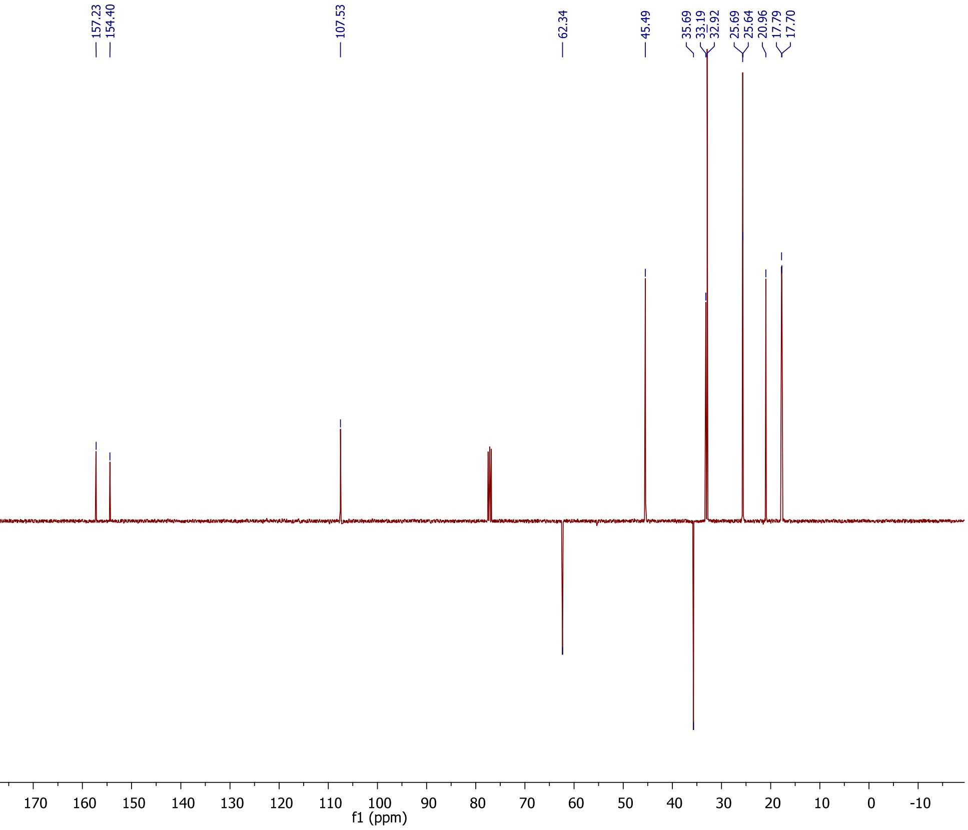
157.23
154.40
107.53
62.34
45.49
35.69
33.19
32.92
25.69
25.64
20.96
17.79
17.70
210 200 190 180 170 160 150 140 130 120 110 100 90 80 70 60 50 40 30 20 10 0 -10
f1 (ppm)

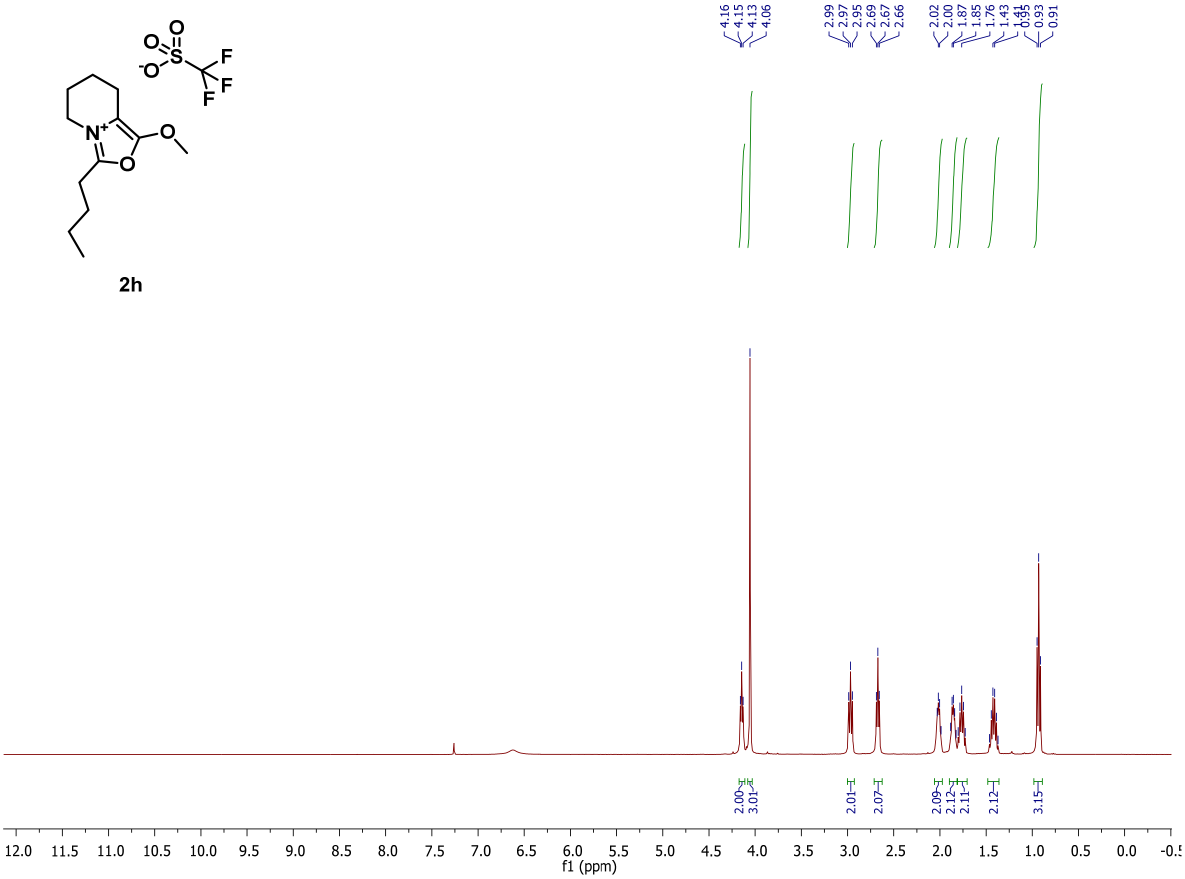
2h
4.16
4.15
4.13
4.06
2.99
2.97
2.95
2.69
2.67
2.66
2.02
2.00
1.87
1.85
1.76
1.43
1.41
0.95
0.93
0.91
2.00
3.01
2.01
2.07
2.09
2.12
2.11
2.12
3.15
f1 (ppm)

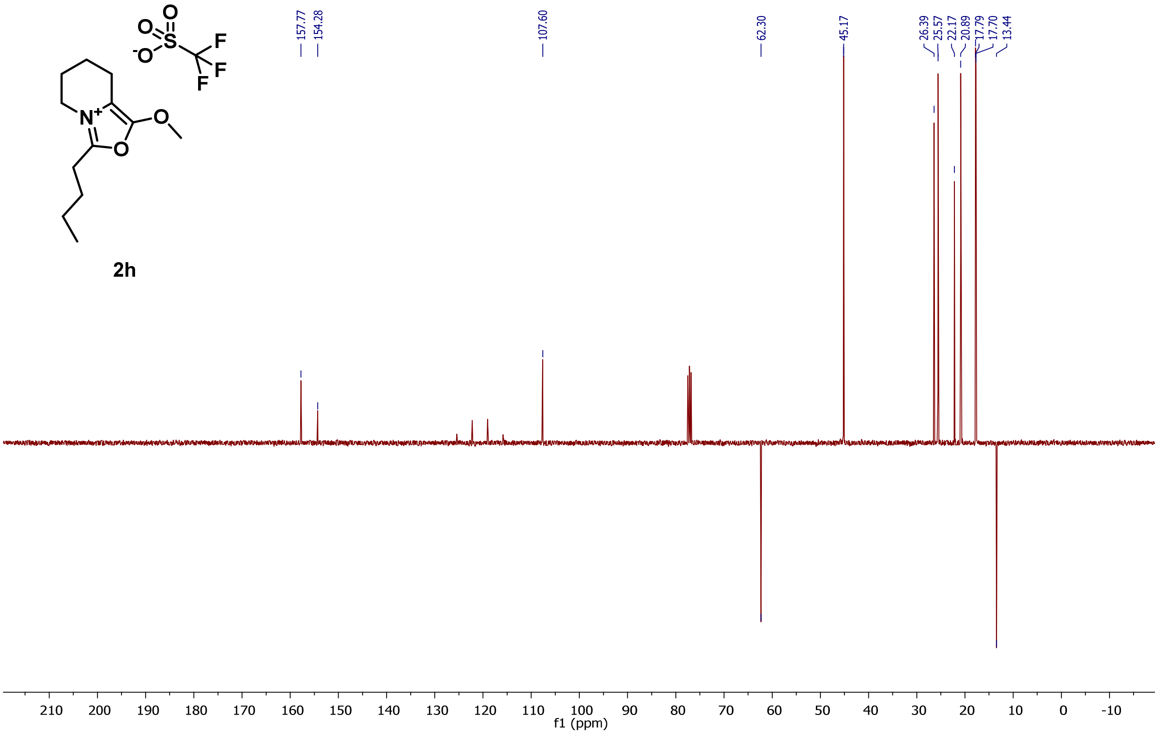
2h
157.77
154.28
107.60
62.30
45.17
26.39
25.57
22.17
20.89
17.79
17.70
13.44
f1 (ppm)

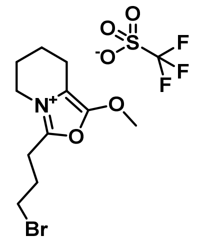
O
O
S
-O
F
F
F
N+
O
O
Br

2i

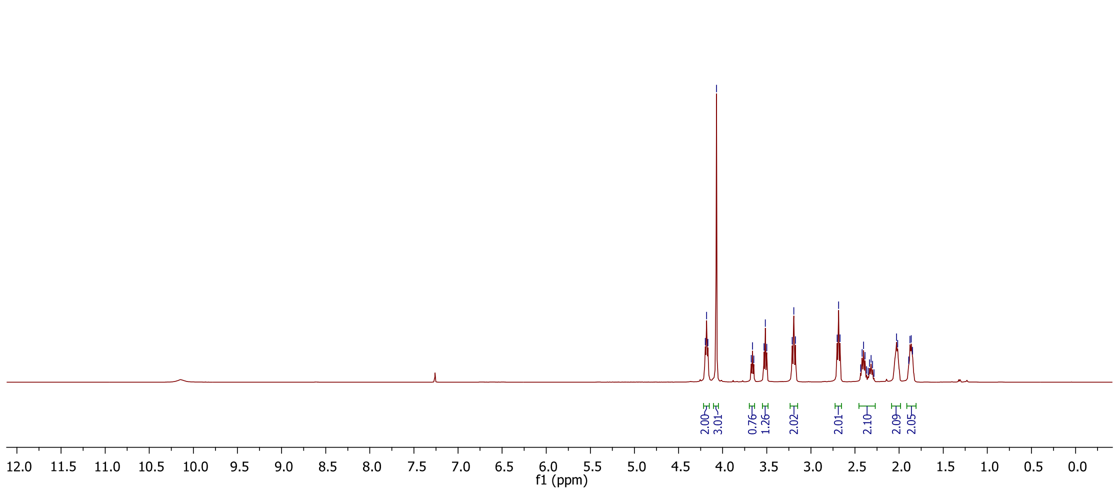
2.00
3.01
0.76
1.26
2.02
2.01
2.10
2.09
2.05
f1 (ppm)

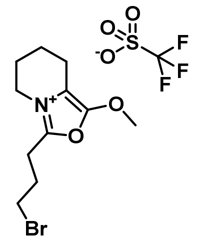
O
O
S
-O
F
F
F
N+
O
O
Br

2i

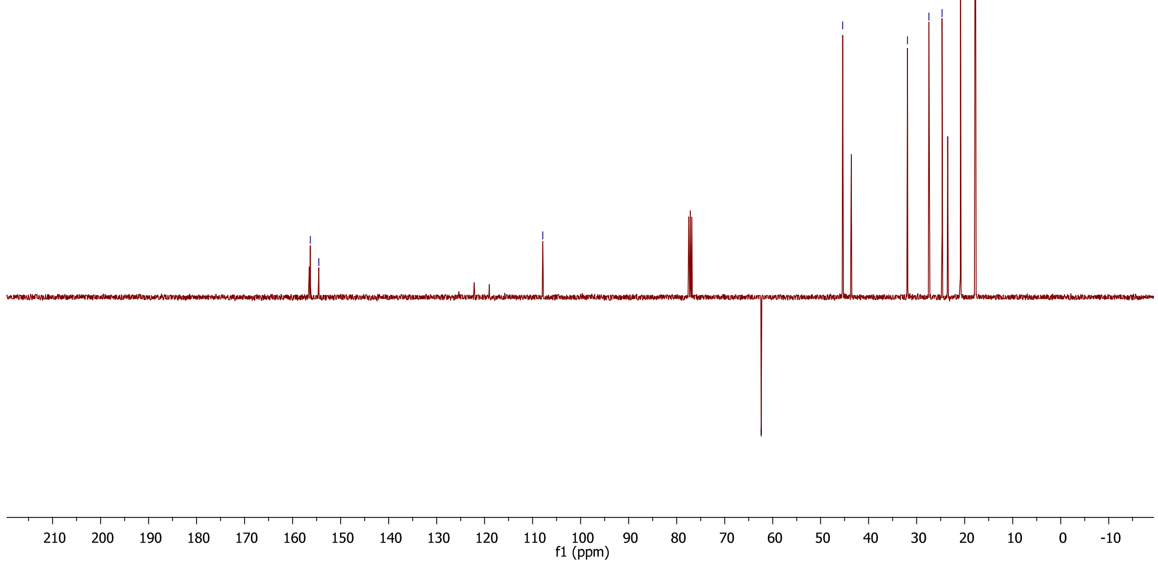
156.30
154.52
107.89
62.39
45.40
31.91
27.42
24.70
20.87
17.82
17.72
f1 (ppm)

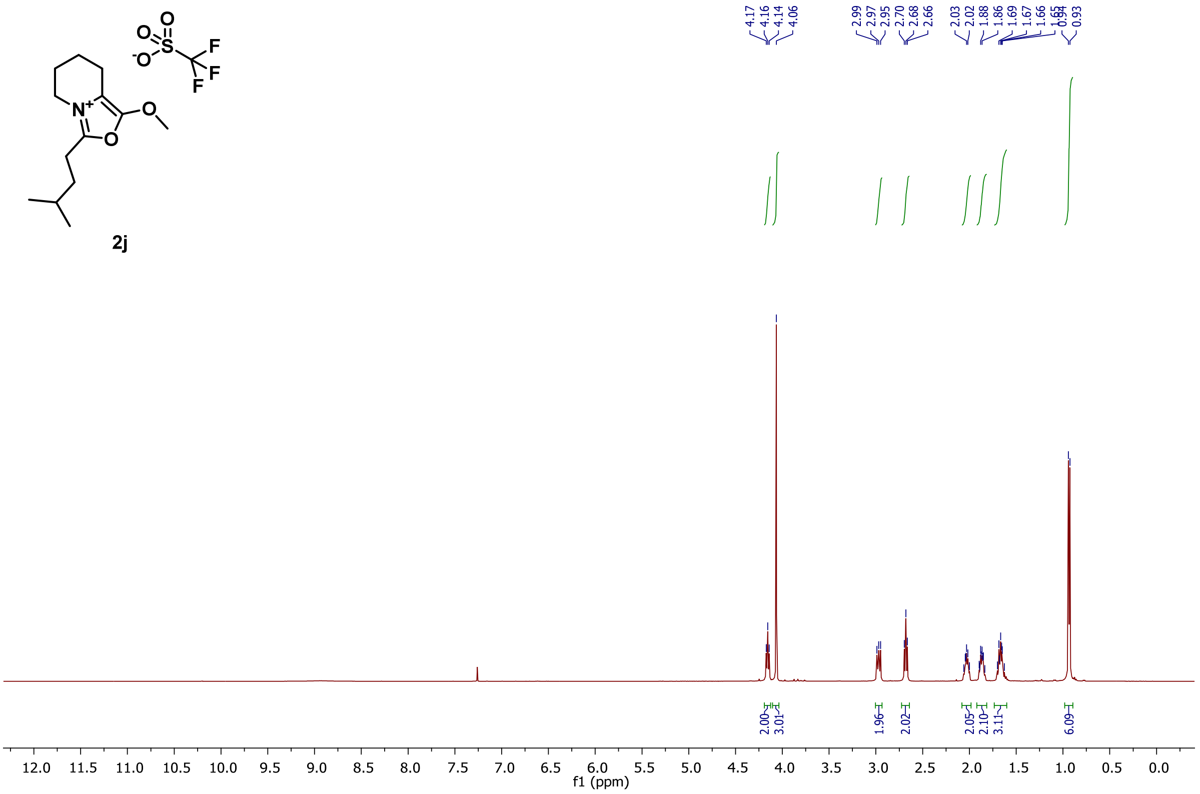
2j
4.17
4.16
4.14
4.06
2.99
2.97
2.95
2.70
2.68
2.66
2.03
2.02
1.88
1.86
1.69
1.67
1.66
1.65
0.94
0.93
2.00
3.01
1.96
2.02
2.05
2.10
3.11
6.09
f1 (ppm)

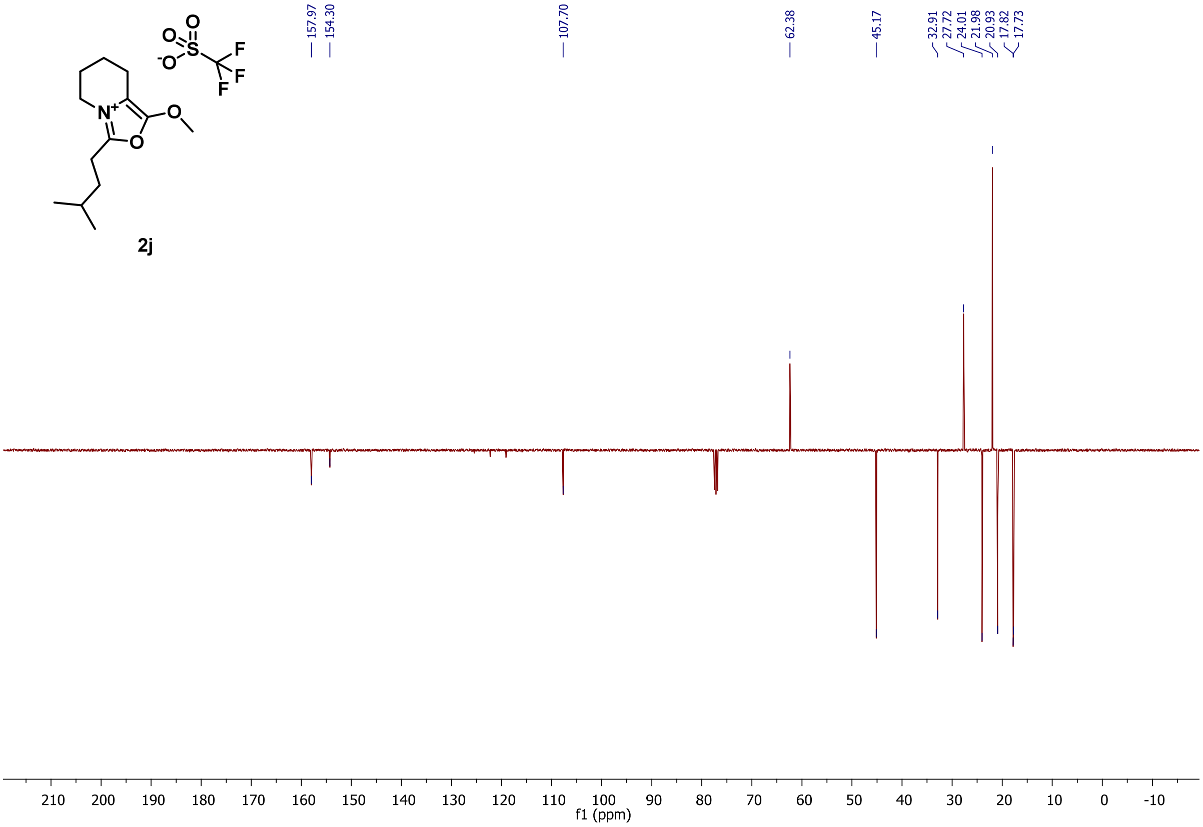
2j
157.97
154.30
107.70
62.38
45.17
32.91
27.72
24.01
21.98
20.93
17.82
17.73
f1 (ppm)

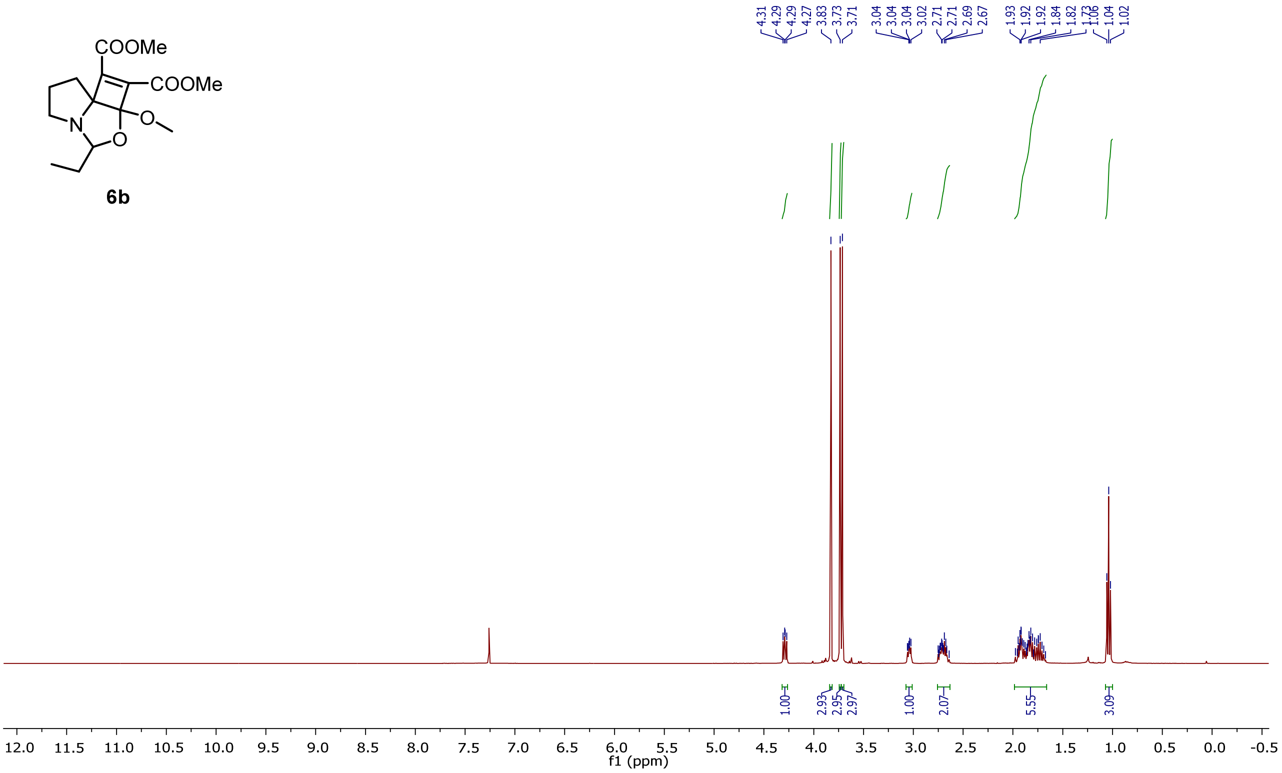
COOMe
COOMe
N
O
O
4.31 4.29 4.29 4.27 3.83 3.73 3.71 3.04 3.04 3.04 3.02 2.71 2.71 2.69 2.67 1.93 1.92 1.92 1.84 1.82 1.73 1.06 1.04 1.02
1.00 2.93 2.95 2.97 1.00 2.07 5.55 3.09
12.0 11.5 11.0 10.5 10.0 9.5 9.0 8.5 8.0 7.5 7.0 6.5 6.0 5.5 5.0 4.5 4.0 3.5 3.0 2.5 2.0 1.5 1.0 0.5 0.0 -0.5
f1 (ppm)

6b

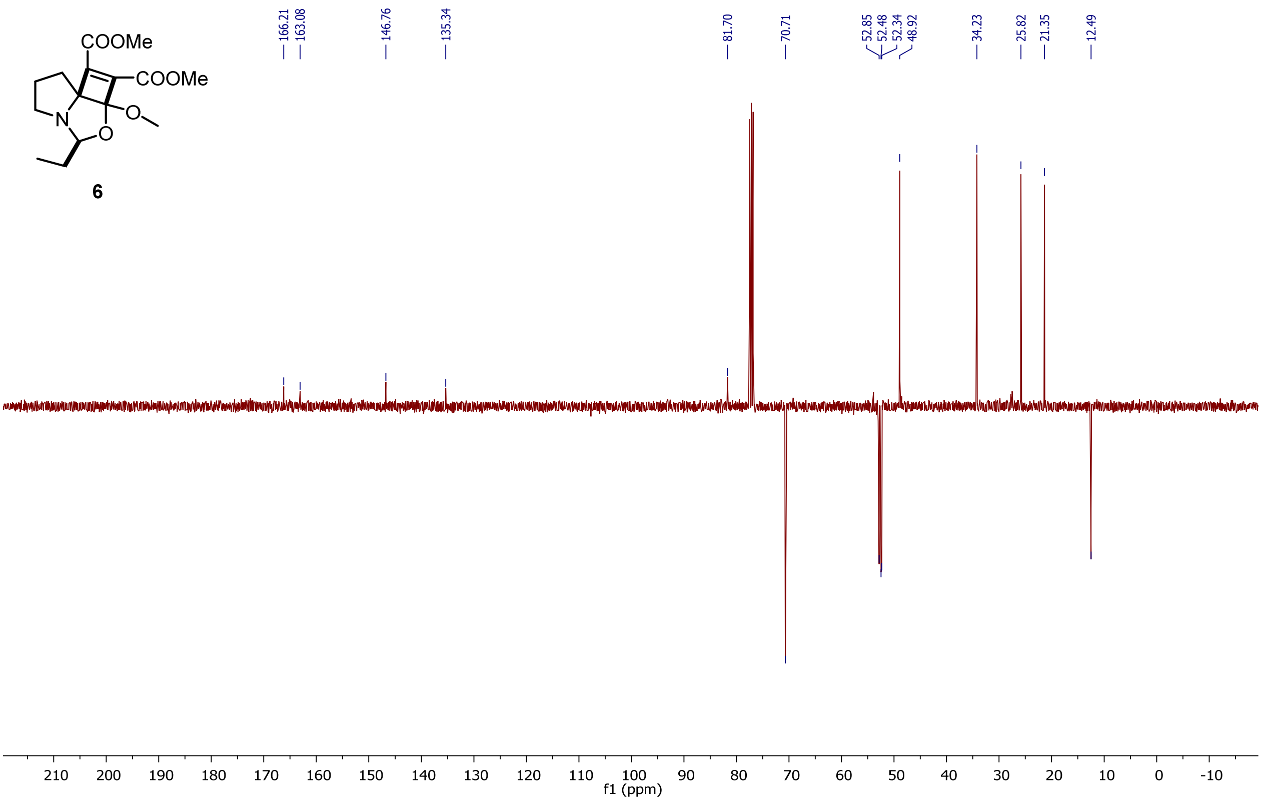
COOMe
COOMe
N
O
O
166.21 163.08 146.76 135.34 81.70 70.71 52.85 52.48 52.34 48.92 34.23 25.82 21.35 12.49
210 200 190 180 170 160 150 140 130 120 110 100 90 80 70 60 50 40 30 20 10 0 -10
f1 (ppm)

6
